# Assessment of exposure risks to COVID-19 among frontline health care workers in Amhara Region, Ethiopia: A cross-sectional survey

**DOI:** 10.1371/journal.pone.0251000

**Published:** 2021-04-29

**Authors:** Seyfe Asrade Atnafie, Demssie Ayalew Anteneh, Dawit Kumilachew Yimenu, Zemene Demelash Kifle

**Affiliations:** 1 Department of Pharmacology, School of Pharmacy, College of Medicine and Health Sciences, University of Gondar, Gondar, Ethiopia; 2 Department of Hospital Clinical Pharmacy, College of Medicine and Health Science, University of Gondar, Gondar, Ethiopia; 3 Department of Pharmaceutics and Social Pharmacy, School of Pharmacy, College of Medicine and Health Sciences, University of Gondar, Gondar, Ethiopia; China University of Mining and Technology, CHINA

## Abstract

**Background:**

The burden to fight with Corona Virus Disease-19 (COVID-19) pandemic has lied to frontline health care workers that are putting themselves at a higher risk in the battle against the disease. This study aimed to assess the exposure health risks of COVID-19 among frontline healthcare workers in the Amhara region, Ethiopia.

**Method:**

A web-based cross-sectional study was conducted on public health workers from May to August 2020. Data were collected using a structured questionnaire via email and telegram services. Both descriptive statistics and bivariate followed by multivariable logistic regression analyses were conducted to identify distribution patterns and factors associated with exposure risks to COVID-19. Odds ratio with 95% Confidence Interval (CI), and a P-value of <0.05 was used to determine statistical significance.

**Result:**

A total of 418 health care workers participated in the study with a response rate of 99.1%. The majority of the study participants 310(74.2%), were males, and 163(39%) were nurses/ midwives respectively. More than half of the respondents 237(56.7%), had reported that they didn`t have face-to-face contact with a confirmed COVID-19 patient. Among the respondents, 173(41.4%), 147(35.2%), 63(15.1%), and 65(15.6%) of the health professionals had always used gloves, medical masks, face shield, or goggles/protective glasses, and disposable gown, respectively. In this study, age between 25–34 years (AOR = 0.20), age between 35–44 years (AOR = 0.13), family size of >6 (AOR = 3.77), work experience of 21–30 years (AOR = 0.01), and good handwashing habit (AOR = 0.44) were the protective factors against COVID-19. On the other hand, perception of non-exposure to COVD 19 (AOR = 9.56), and poor habit of decontamination of high touch areas (AOR = 2.52) were the risk factors associated with confirmed COVID 19 cases among health care workers.

**Conclusion:**

Poor adherence to personal protective equipment use and aseptic practices during and after health care interactions with patients were identified. Strategies should be implemented to institute effective and sustainable infection control measures that protect the health care workers from COVID-19 infection.

## Introduction

The etiology for coronavirus disease (COVID-19) is a novel human coronavirus (SARS-COV-2) the first case of which was first reported in Wuhan, China, in December 2019 [1]. The first case of COVID-19 appeared in China, in December 2019. The World Health Organization (WHO) declared it a pandemic in march, 2020 [2]. As of October 26, 2020, more than 42,745,212 confirmed cases of COVID-19 have been registered by WHO worldwide with nearly 1,150,961 deaths. Currently, 10,311,358 patients are infected by COVID-19 [3, 4]. The symptoms for COVID-19 infection include loss of taste and smell, dry cough, shortness of breath, fever, and pneumonia that might be mild to severe in severity [5]. It is supposed that the incubation period of Coronavirus infection is 2–14 days and symptoms typically appear between these days [6].

Globally, many countries had various forms of restriction to prevent morbidity and mortality due to COVID-19 [7]. However, only some countries were effective to halt the spread of the disease in which many developing countries including Sub-Saharan African countries failed to stop the spread of disease transmission [8]. The burden to fight the COVID-19 pandemic lied to frontline health care workers. Health care providers are putting themselves at high risk in the battle against COVID-19. Many healthcare workers were infected and died with COVID-19, and many of them were quarantined to prevent the spreading of the infection [9].

COVID-19 is a higher risk for health workers who work in critical care, emergency medicine, infectious diseases, pulmonary medicine departments, and other departments. Hand hygiene, proper handwashing, and Personal protective equipment are critical in reducing the spread of coronavirus infection in health facilities and communities. Thus, adequate training and resources are required for health professionals to prevent cross-contamination to other patients who receive care in health facilities [10–12].

Front line health care workers directly face the COVID-19 pandemic and had higher exposure to health risks such as psychological distress, pathogen, occupational burnout, fatigue, stigma, physical violence, and long working hours [13]. According to Amnesty International, over 7,000 health care workers have lost their lives around the world after contracting COVID-19, a double burden in the fight against COVID-19. Poor understanding and bad practices of COVID-19 preventing measures among health workers can result in delayed identification and treatment leading to the rapid spread of coronavirus infections [14]. The guiding principle for healthcare workers and online courses has been established by WHO, to improve the knowledge and prevention strategies [15]. Even though addressing the knowledge and practice gaps of frontline health workers is a high priority, there is a lack of prospective data to inform such efforts, especially in developing countries [16, 17]. Data related to exposure health risk and factors related to this are also limited [18]. Thus, this study aimed to assess the exposure risk of COVID-19 among frontline health workers in the Amhara region, Ethiopia.

## Methods

### Ethical consideration

Ethical clearance was obtained from the research ethics committee of the school of pharmacy, University of Gondar with a Reference number of So/P-784-2020, and the study was also conducted following the Declaration of Helsinki. Informed written consent, was obtained from the study participants at the beginning of filling the survey. The purpose of the study was explained in written words in the introduction part of the survey. The respondents were notified that they had the right to refuse or stop at any point in the data collection. The information collected from respondents was kept confidential and there were no personal identifiers in the questionnaire.

### Study design and settings

An institution-based cross-sectional study was done from 01, May to 31^st^, august 2020 among healthcare workers working in government hospitals and health centers of the Amhara Region, Ethiopia. Amhara Region is the 2^nd^ biggest region in Ethiopia constituting twelve administrative zones. It is located in the Northwest part of the country and Bahir Dar is the capital city of the region. In 2018, it was reported that there were 77 hospitals, 3342 health posts, 4267 public health facilities, 848 health centers, and a total of 38,000 healthcare workers in the region that provide healthcare services to a total population of 21,841, 999 (4,089,997 urban and 17,752,002 rural) [19].

### Study participants

Health care professionals, including doctors, pharmacists, nurses, health officers, laboratory technicians, and patient transporters working in government hospitals and health centers of the Amhara Region, Ethiopia, were included in this survey. Medical students and Healthcare professionals presently not working in government hospitals and health centers or previously participated in a similar study concerning COVID-19 were excluded.

### Sample size determination and sampling procedures

The sample size of the respondents was determined via a single population proportion formula based on the following assumption: Since there is no previous study in Ethiopia, the maximum prevalence was used, P = 0.5, 95% confidence level, 5% margin of error, and 10% non-response rate was added and the required sample size became 422. Respondents were selected from five hospitals and ten health centers using a simple random sampling technique.

### Data collection tools and techniques

Data were collected using online data collection tools, e-mail, and social media platforms of health care workers in government hospitals and health centers of the Amhara Region, Ethiopia. The tool was adopted and modified into the local context from WHO Interim Guidance [20]. The questionnaire was prepared in English and translated into Amharic (local language) by experts in both languages. The responses were back-translated into English for rechecking of meanings and concepts. The questionnaire has three parts: Health worker background information, assessment of exposure to COVID-19, and adherence to infection prevention and control (IPC) during health care interactions. The validity of the questionnaire was evaluated by pretesting twenty-one healthcare workers from health facilities out of the study area. Based on the results of the pretest, we removed the IPC in isolation centers part in the WHO interim guidance. The completeness and accuracy of the collected data were checked daily by the supervisors.

### Data quality assurance

The questionnaire was designed with ease of use and pretested with 5% of the sample size before data collection. Only eligible respondents were allowed to participate in the online survey. Health workers were informed on how to complete and sent the questionnaire. The supervisor regularly sent a remark to respondents so that they had filled and returned the questionnaire and supervised and followed the returned questionnaires for completeness and consistency of the data.

### Data processing and analysis

The returned data were transferred to SPSS version 24 for analysis. Descriptive statistics like frequency, percentage, mean, standard deviation, and median were used for data analysis. Figures, tables, and texts were used to summarize the descriptive statistics of the study. All continuous variables were checked for normality using the Hosmer-Leme show goodness of fit test. Bivariate and multivariable logistic regression analyses were applied to determine factors associated with the exposure risk of COVID-19. Odds ratio (OR) with 95% CI was used to assess the strength of association, and p-value <0.05 was used to determine the statistical significance of health risk of COVID-19 among frontline health care workers.

## Result

### Sociodemographic characteristics of respondents

A total of 418 subjects were enrolled in the study with a response rate of 99.1%. The majority of the study participants were male 310 (74.2%), hospital workers 328(78.5%), that had a work experience of between 1 and 10 years 306 (73.2%). About 262(62.7%) of respondents were BSc degree holders, and 163(39%) of them were nurses/ midwives by profession. Concerning marital status, more than half of the study participants 216 (51.7%) were single and have a family size of less than 3 (50%) (Table 1).

**Table 1:** Socio-demographic characteristics of health workers in the Amhara region, 2020 (N = 418).

Variables		Frequency	Percentage
**Sex**	Male	310	74.2
	Female	108	25.8
**Age group**	18–24	52	12.4
	25–34	285	68.2
	35–44	70	16.7
	45–54	11	2.6
**Marital status**	Single	216	51.7
	Married	190	45.5
	Separated	12	2.9
**Type of health care setting**	Hospital	328	78.5
	Outpatient clinic	11	2.6
	Health center	41	9.8
	Home care	14	3.3
	Community pharmacy	24	5.7
**Family size**	<3	209	50
	3–5	148	35.4
	>6	61	14.6
**Education status**	Diploma	73	17.5
	BSc	262	62.7
	MSc	57	13.6
	PhD/Specialist	12	2.9
	Other*	14	3.3
**Experience**	<1	29	6.9
	1–10	306	73.2
	11–20	68	16.3
	21–30	15	3.6
**Health care facility unit type**	Outpatient	76	18.2
	Emergency	92	22.0
	Medical unit	97	23.2
	Laboratory	20	4.8
	Pharmacy	67	16.0
	Quarantine center	14	3.3
	Other**	52	12.4
**Type of health work**	Medical doctor	83	19.9
	Nurse/Midwife	163	39.0
	Patient transporter	14	3.3
	Health officer	19	4.5
	Pharmacy Personnel	77	18.4
	Laboratory Personnel	22	5.3
	Other***	40	9.6

*Other includes primary school, secondary school, certificate.

**Other includes radiology, intensive care unit, cleaning service, ambulance.

***Other includes radiology/x-ray technician, admission clerk, cleaner, driver.

### Assessment of exposure status of health professionals for COVID 19

Of the surveyed health workers, over half, 237(56.7%), of the health professionals didn`t have face-to-face contact (within 1 meter) with a confirmed COVID-19 patient. On the other hand, about 260(62.2%) of the participants reported that they had no direct contact with the environment where the confirmed COVID-19 patient was cared for. The majority, 375 (89.7%) of the health professionals were not present when an aerosol-generating procedure was performed. Regarding exposure status; about 78(18.7%) of the study participants have reported that they had confirmed exposure to COVID-19 (Table 2).

**Table 2:** Assessment of exposure status of health professionals for COVID 19 in Amhara region, 2020 (N = 418).

Variables		Frequency	Percentage
Provide direct care to a confirmed COVID-19 patient	Yes	75	17.9
	No	257	61.5
	Unknown	86	20.6
face-to-face contact (within 1 meter) with a confirmed COVID-19 patient	Yes	91	21.8
	No	237	56.7
	Unknown	90	21.5
direct contact with the environment where the confirmed COVID-19 patient was cared for	Yes	89	21.3
	No	260	62.2
	Unknown	69	16.5
Present when any aerosol-generating procedure was performed	Yes	43	10.3
	No	375	89.7
Exposure status	Exposed	164	39.2
	Not exposed	167	40
	Probably exposed	87	20.8
Confirmed exposure	Yes	78	18.7
	No	340	81.3

### Adherence to personal protective equipment use during health care interactions

In this study, about 173(41.4%), 147(35.2%), 63(15.1%), and 65(15.6%) of the study participants had always used gloves, medical masks, face shields, or goggles/protective glasses, and disposable gown, respectively. On the contrary, 6(1.4%), 10(2.4%), 84(20.1%), and 99(23.7%) of the participants hadn’t used at all gloves, medical mask, face shield, or goggles/protective glasses, and disposable gown, respectively. In the present study, 194(46.4%) of the health care workers had good personal protective equipment use habits against COVID 19 protection (Table 3).

**Table 3:** Adherence to personal protective equipment use during health care interactions in the Amhara region, 2020 (N = 418).

Personal protective equipment (PPE) use	Always, as recommended	Most of the time	Occasionally	Rarely	Not used
**Single gloves**	173(41.4)	98(23.4)	67(16.0)	74(17.7)	6(1.4)
**Medical mask**	147(35.2)	93(22.2)	66(15.8)	102(24.4)	10(2.4)
**Face shield or goggles/protective glasses**	63(15.1)	73(17.5)	32(7.7)	166(39.7)	84(20.1)
**Disposable gown**	65(15.6)	52(12.4)	27(6.5)	175(41.9)	99(23.7)
**Personal protective equipment use**	Good*	194 (46.4)			
	Bad*	224 (53.6)			

*Good personal protective equipment use is added response of health workers which is greater than mean value considering always as 5, mostly as 4, occasionally as 3, rarely as 2 and not used as 1.

*Bad personal protective equipment use is added response of health workers which is less than mean value considering always as 5, mostly as 4, occasionally as 3, rarely as 2 and not used as 1.

### Adherence to handwashing practice during health care interactions

In the current study, 163(39.0%), 174(41.6%), 198(47.4%), and 184(44.0%) of the respondents always washed their hands after touching patients with COVID-19, after a clean or aseptic procedure was performed, after exposure to body fluid, and after touching the COVID-19 patient’s surroundings (bed, door handle, etc), respectively. However, 26(6.2%), 21(5.0%), 26(6.2%), and 24(5.7%) of the health care workers didn’t wash their hands at all after touching patients with COVID-19, after a clean or aseptic procedure was performed, after exposure to body fluid, and after touching the COVID-19 patient’s surroundings, respectively. In general, over half of health care workers (56.7%) had good handwashing practices (Table 4).

**Table 4:** Adherence to handwashing practice during health care interactions in the Amhara region, 2020 (N = 418).

Handwashing practice	Always, as recommended	Most of the time	Occasionally	Rarely	Not used
After touching patients with COVID-19	163 (39.0)	78 (18.7)	58 (13.9)	93(22.2)	26(6.2)
After clean or aseptic procedure was performed	174 (41.6)	91 (21.8)	51 (12.2)	81 (19.4)	21 (5.0)
After exposure to body fluid	198 (47.4)	88 (21.1)	33 (7.9)	73 (17.5)	26 (6.2)
After touching the COVID-19 patient’s surroundings	184 (44.0)	85(20.3)	51(12.2)	74(17.7)	24(5.7)
Handwashing practice	Good*	237(56.7)			
	Bad*	181(43.3)			

*Good handwashing practice is added response of health workers which is greater than mean value considering always as 5, mostly as 4, occasionally as 3, rarely as 2 and not used as 1.

*Bad handwashing practice is added response of health workers which is less than mean value considering always as 5, mostly as 4, occasionally as 3, rarely as 2 and not used as 1.

### Factors associated with confirmed COVID 19 cases among health care workers

In the present study, 78 (18.66%) had reported that they have had a confirmed COVID-19 infection. Based on the multivariable logistic regression model, health care workers between the ages of 25–34 years were 80 times less likely to be infected with COVID-19 infection than respondents whose ages were between 18–24 years (AOR = 0.20, 95% CI = 0.041–0.96). Similarly, health care workers between 35–44 years of age were 87 times less likely to be infected with COVID-19 infection than respondents aged 18–24 years (AOR = 0.13, 95% CI = 0.02–0.86). Health workers who had > 6 family size were nearly 4 times more likely to be infected with COVID-19 infection compared to health care workers who had < 3 family size (AOR = 3.77, 95% CI = 1.07–13.26). Health care workers who had 21–30 years of work experience were found to be less likely to be infected with COVID-19 as compared to health care workers who had < 1 year of work experience (AOR = 0.01, 95% CI = 0.01–0.06).

Health workers who perceived as if they will not be exposed to COVD 19 infection were nearly ten times more likely to be infected with COVD 19 compared to health care workers who perceived they will be exposed to COVD 19 infection (AOR = 9.56, 95% CI = 3.51–26.06). Respondents who had good handwashing habits were 56 times less likely to be infected with COVID-19 infection compared to those who had bad handwashing habits (AOR = 0.44, 95% CI = 0.20–0.95). Health workers who work in an institution without a habit of decontamination of high touch areas were 2.5 more likely to be infected with COVID-19 as compared to institutions with a habit of decontamination of high touch areas (AOR = 2.52, 95% CI = 1.12–5.65). There was no significant association between personal protective use by health workers and exposure to COVID-19 (Table 5).

**Table 5:** Bivariate and multivariate logistic regression of confirmed COVID 19 cases and associated factors in the Amhara region, 2020 (N = 418).

Variables	COVID 19		COR (CI = 95%)	AOR (CI = 95%)	P-Value
	Yes	No			
**Age**					
18–24	17(21.8)	35(10.3)	1	1	
25–34	51(65.4)	234(68.8)	2.23(1.16–4.23)	0.20(0.04–0.96)*	**0.044**
35–44	9(11.5)	61(17.9)	3.29 (1.33–8.17)	0.13(0.02–0.86)*	**0.034**
45–54	1(1.3)	10(2.9)	4.86 (0.57–41.11)	1.25(0.06–27.12)	0.889
**Marital status**					
Single	50(64.1)	166(48.8)	1	1	
Married	26(33.3)	164(48.2)	1.90(1.13–3.20)	1.54(0.61–3.90)	0.360
Separated	2(2.6)	10(2.9)	1.51(0.32–7.10)	0.194(0.02–1.86)	0.155
**Type of health care setting**					
Hospital	68(87.2)	260(76.5)	1	1	
Outpatient clinic	1(1.3)	10(2.9)	2.62(0.33–20.78)	1.95(0.15–25.26)	0.608
Health center	6(7.7)	35(10.3)	1.53(0.62–3.78)	0.847(0.22–3.29)	0.811
Home care	2(2.6)	12(3.5)	1.57 (0.34–7.18)	2.17(0.28–17.08)	0.461
Community pharmacy	1(1.3)	23(6.8)	6.02(0.79–45.34)	3.53(0.20–59.49)	0.382
**Family size**					
<3	17	38	1	1	
3–5	19	93	2.55(1.41–4.63)	1.77(0.73–4.23)	0.201
>6	35	143	1.91(0.88–4.15)	**3.77(1.07–13.26)**	**0.038**
**Education level**					
Diploma	12(15.4)	61(17.9)	1		
BSc	52(66.7)	210(61.8)	0.79(0.39–1.58)	0.89(0.30–2.63)	0.834
MSc	8(10.3)	49(14.4)	1.21(0.46–3.18)	0.89(0.20–3.80)	0.869
PhD/Specialist	4(5.1)	8(2.4)	0.39(0.10–1.52)	0.43(0.06–2.97)	0.390
Other*	2(2.6)	12(3.5)	1.18(0.23–5.96)	1.74(0.21–14.41)	0.610
**Experience**					
<1	46(59.0)	260(76.5)	1	1	
1–10	5(6.4)	10(2.9)	0.354(0.12–1.08)	0.45(0.09–2.24)	0.351
11–20	5(6.4)	63(18.5)	2.23(0.85–5.84)	1.83(0.52–6.48)	0.412
21–30	22(28.2)	7(2.1)	0.06(0.02–0.14)	0.01(0.01–0.06)*	**0.013**
**Health care facility unit type**					
Outpatient	16(20.5)	60(17.6)	1	1	
Emergency	33(42.3)	59(17.4)	0.477 (0.24–0.96)	0.81(0.25–2.51)	0.711
Medical unit	16(20.5)	81(23.8)	1.35(0.63–2.91)	1.38(0.44–4.33)	0.577
Laboratory	7(9.0)	15(4.4)	0.57(0.19–1.64)	0.13(0.01–12.86)	0.388
Pharmacy	1(1.3)	65(19.1)	17.33(2.23–34.71)	2.51(0.14–45.66)	0.534
Quarantine center	1(1.3)	13(3.8)	3.47(0.42–28.52)	3.42(0.30–38.64)	0.320
Other**	4(5.1)	47(13.8)	3.13(0.98–9.99)	5.04(0.85–29.59)	0.073
**Type of health work**					
Medical doctor	36(46.2)	47(13.8)	1	1	
Nurse/Midwife	26(33.3)	133(39.1)	3.92 (2.14–7.17)	2.43(0.77–7.66)	0.130
Patient transporter	3(3.8)	13(3.8)	3.32 (0.88–12.53)	0.77(0.11–5.45)	0.789
Health officer	2(2.6)	18(5.3)	6.89 (1.50–31.65)	4.41(0.60–32.32)	0.145
Pharmacy Personnel	1(1.3)	74(21.8)	56.68(7.52–27.4)	14.17(0.94–13.67)	0.055
Laboratory Personnel	7(9.0)	18(5.3)	1.97 (0.74–5.22)	6.96(0.06–68.15)	0.419
Other***	3(3.8)	37(10.9)	9.45 (2.69–33.11)	7.68(0.22–18.27)	0.128
**Perceived exposure status**					
Perceived exposed	46(59.0)	118(34.7)	1	1	
Perceived non exposed	13(16.7)	154(45.3)	4.618 (2.39–8.94)	9.56(3.51–26.06)*	0.001
Perceived probably exposed	19(24.4)	68(20.0)	1.40(0.76–2.57)	2.75(0.95–7.99)	0.063
**Personal protective equipment uses**					
Good	31(39.7)	149(43.8)	1.18(0.72–1.95)	1.03(0.47–2.26)	0.938
Bad	47(60.3)	191(56.2)	1	1	
**Handwashing habit**					
Good	52(66.7)	184(54.1)	0.59(0.35–0.99) *	0.44(0.20–0.95)*	**0.036**
Bad	26(33.3)	156(45.9)	1	1	
**Decontamination of high touch areas**					
Yes	58(74.4)	213(62.6)	1	1	
No	20(25.6)	127(37.4)	1.73(0.99–3.01)	2.52(1.12–5.65)*	**0.025**
**Changing of wet medical masks**					
Yes	49(62.8)	251(73.8)	1.67(0.99–2.81)	0.51(0.18–1.39)	0.191
No	29(37.2)	89(26.2)	1	1	
**Training need**					
Yes	35(44.9)	94(27.6)	2.13 (1.28–3.53)	1.08(0.44–2.66)	0.868
No	43(55.1)	246(72.4)	1	1	

N.B.

* = p-value<0.05

**p-value<0.01

***p-value<0.001, COR = crude odds ratio; AOR = adjusted odds ratio.

## Discussion

COVID-19 infection is still a rapidly spreading global health problem affecting all sectors [21]. Health care workers acquired COVID-19 infection at a higher rate than the general population [21–24]. In the present study, 18.66% had reported that they have had a confirmed COVID-19 infection. Previous studies conducted in Italy, Netherlands, and the United Kingdom reported that the prevalence rate of COVID-19 among health care workers was 3%, 9%, and 18%, respectively [25–27]. As such, health care workers working throughout the world should have satisfactory knowledge about all features of the disease such as established prevention strategies, proposed treatment, diagnosis, and clinical manifestation. To the best of the author’s knowledge, this is the first study in Ethiopia that assessed the exposure health risk of COVID-19 among frontline healthcare workers in the Amhara region, Ethiopia. Besides, there are also very limited studies regarding the exposure health risk of COVID-19 among frontline health workers globally.

In the current study, the mean age of the respondents was 33 years and there was a statistically significant difference among different age groups. Health care workers whose ages were between 25–34 years were 80 times less likely to be infected with COVID-19 infection than respondents whose ages were between 18–24 years. Similarly, health care workers between 35–44 years of age were 87 times less likely to be infected with COVID-19 infection than respondents aged 18–24 years. A study done on health professionals in the USA revealed that the mean age of health care workers being affected with COVID-19 was 42 years [28]. A similar study conducted in China showed that the mean age of the affected health professionals was 37 years [29]. According to a study conducted in Bangladesh, health professionals were affected by COVID-19 infection at a fairly younger age. However, in China, relatively older-aged health professionals were affected and the age variance among health care workers was significant [29].

The current finding showed that the likelihood of being infected with COVID-19 was higher among health care workers working in the Emergency ward though the difference was not statistically significant. However, a study conducted in Bangladesh showed that the possibility of being infected with COVID-19 was higher among health care workers working in the Intensive Care Unit (ICU), though, the difference was not statistically significant [24]. Similarly, a study done in Wuhan, China revealed that health professionals working in the ICU had two times more likelihood of getting infected with COVID-19 than health professionals working in the general wards [29]. This difference might be due to the dynamic nature of health workers that health workers are not specialized in a single ward and work in the form of rotation. For the possible prevention of COVID-19 spread, health workers should specialize in specific duties and should permanently work until the COVID-19 infection spread is controlled. The prevention of COVID-19 infection in the workplace should be applied by integrating the information based on the degree of the spreading risk of this virus in different areas of health facilities, determined by its location concerning the areas of greatest risk and by the type of work carried out, as recommended by the guidance on preparing workplaces for COVID-19 infection [30, 31].

In this study, 6(1.4%), 10(2.4%), 84(20.1%), and 99(23.7%) of the participants were not using gloves, medical masks, face shields, or goggles/protective glasses, and disposable gown, respectively. Thus, this finding stress adequate supply and proper use of personal protective equipment, which are of the greatest role in preventing COVID-19 infection among health care workers in the Amhara region. Previous studies advised taking appropriate Personal Protective Equipment (PPE) measures throughout direct patient care and performing aerosol generated procedures until the health care workers assured the patient is free from COVID-19 infection, particularly in the current pandemic condition. According to World health organization recommendations, the use of N95 masks exhibited a protective factor against COVID-19 infection among health care workers who performed the aerosol-generated procedure. Proper use of goggles and face shields significantly protected the health care workers from COVID-19 infection [24, 32].

Among health professionals with COVID-19, 21.8% reported close contact with a person with COVID-19, which is lower than previous reports [33, 34]. The pre-symptomatic or asymptomatic transmission of the COVID-19 infection through respiratory droplets was reported in the previous study [35]. WHO and CDC contemplate the transmission of the disease with particles > 5 microns as a transmission via droplets, whereas in the case of the size of <5 microns as an aerosol transmission [36]. The conjunctiva is vulnerable to the entrance of microorganisms. Thus, it is crucial to protect the eyes from exposure to COVID-19 infection when there is close contact with patients infected with COVID-19 infection [30].

Proper handwashing practice is a very important measure to prevent the transmission and spread of COVID-19 infection. Hands should be washed with water and soap for about 40–60 seconds; if water and soap are not obtainable, a 62%–71% alcohol-based sanitizer is recommended [30]. Respondents who had good handwashing habits were 56 times less likely to be infected with COVID-19 infection compared to those who had bad handwashing habits. However, health professionals who haven’t had a habit of decontamination of high touch areas were 2.5 more likely to be infected with COVID-19 as compared to health care workers who had a good habit of decontamination of high touch areas. In a previous study done in the Jugal hospital, East Ethiopia, the habit of handwashing practice after touching body fluids, blood, and secretions was 100% and after doing a procedure was 74.1. However, it is only 6.63% of respondents wash their hands before a procedure [37]. Similarly, another study revealed that contact with a patient’s body fluid was the common motive for always washing hands (87.3%) [38]. However, the present study showed poor handwashing practice (56.7%). Therefore, proper handwashing practices should be improved in protecting health workers from acquiring COVID-19 infection.

Health care workers who had > 6 family size were nearly 4 times more likely to get infected with COVID-19 infection compared to health care workers who had < 3 family size. To reduce the transmission and spread of COVID-19 infection, safety measures must be applied to stay at home once exposed to patients infected with COVID-19, besides keeping sanctions to wear masks, physical distancing, and wash hands. In the condition when a family member or close contact is infected with COVID-19 infection, extra prevention techniques should be applied to decrease the transmission, such as wearing gloves, reducing shared items and meals, wearing masks, and disinfecting and cleaning the home, for those with and without known COVID-19 infection [34]. Though, implementing such recommendations is difficult in families having large family sizes.

Another interesting finding of the current study was that health care workers who perceived they will not be exposed to COVD 19 infection were nearly ten times more likely to be infected with COVD- 19 compared to health care workers who perceived they will be exposed to COVD 19 infection. This could be because those health professionals who perceive that they will not be exposed to the infection may become less cautious and thus increase their probability of being exposed and infected with the virus as compared to their counterparts who will become more cautious and careful to avoid any chance of exposure/ infection. Continuous assessment of numerous kinds of exposures and activities as health care workers is crucial. Exposures and activities should consider the use of masks and social distancing anywhere in the health facilities since it could be a risk factor for SARS-CoV-2 infection [24]. Implementing safe practices to decrease exposures to COVID-19 infection during drinking, on-site eating, and health care works in health facilities should be considered to protect health care workers and slow the spread and transmission of COVID-19 infection.

A previous study revealed that decontamination of hospital surroundings plays a significant role in reducing infection rates among health professionals [39]. The dearth of control of environmental decontaminants and insufficient infection control and prevention measures could be attributed to infection [39]. Health professionals who haven’t had a habit of decontamination of high touch areas were 2.5 more likely to be infected with COVID-19 as compared to health care workers who had a habit of decontamination of high touch areas.

In this study, health care workers who had < 1 year of work experience were more likely to be infected with COVID-19 as compared to health care workers who had 21–30 years of work experience. This may be because more experienced health care professionals have good practice in following guidelines recommendations particularly the use of personal protective equipment as it is apparent that the use of PPE could be supportive in dropping the transmission and spread of COVID-19 infection in health facilities [40].

## Limitation of the study

One of the limitations is bias occurred as a result of study design (cross-sectional) since much less appropriate to determine definitive cause and effect associations. As the study design is cross-sectional and depends on self-reported assessment, under-or over-reporting is very likely. It was also based on online data collection techniques using social media platforms and email. People may give wrong information in the online data collection modalities which can`t be checked in this study. There is also a probability that health workers may be exposed to COVID-19 from community transmission which can`t be ruled out from this study.

## Conclusion

The current study had tried to reveal the current challenges/ exposure health risks which health care professionals in the developing world are facing towards the battle against COVID-19 infection. A significant rate of COVID-19 infection among health care providers was observed. Poor adherence to personal protective equipment uses and aseptic practices during and after health care interactions with patients were identified. Age, family size, years of work experience, Perception towards COVID-19 exposure, handwashing habit, and habit of decontamination of high touch areas were factors associated with confirmed COVID-19 cases among health care workers. Strategies should be implemented to institute effective and sustainable infection control measures that protect the health care workers from COVID-19 infection through psychological support, incentives, availability of personal protective equipment, education/ training, and readiness of staff.

## Supporting information

S1 File This is questionnaire used for the study (English). (DOCX)

S2 File This is questionnaire used for the study (Amharic.). (DOCX)

S3 File This is SPSS data. (DOCX)
