# Supplementary material for: Assessment of exposure risks to COVID-19 among frontline health care workers in Amhara Region, Ethiopia: A cross-sectional survey
Source: PLoS One. 2021 Apr 29;16(4):e0251000. doi: 10.1371/journal.pone.0251000 (PMC8084207; doi:10.1371/journal.pone.0251000)
Supplement: S1 File — (DOCX) [file pone.0251000.s001.docx]

**University of Gondar**

**College of Medicine and Health Sciences, School of pharmacy**

**Consent Information sheet**

My name is __________________________. I am here on behalf of Gondar University research group staffs. We are conducting a research on **‘Assessment Exposure Risks to COVID-19 Among Frontline Health care Workers in Amhara Region, Ethiopia: A cross-sectional survey’** Their research project is approved by the Research Ethics committee of Gondar University. You are selected randomly to participate in this study. Your participation is purely based on your willingness. You have the right to choose not to take part in this study. If you choose to take part, you have the right to stop at any time. If you are willing to participate or refuse or decide to withdraw later, you will not be subjected to any ill-treatment.

If you agree to participate in the study, you will be asked to answer some questions regarding to exposure risks to COVID-19. The interview with you will take about 20 minutes. This study aimed to assess the exposure health risks of COVID-19 among frontline healthcare workers in the Amhara region, Ethiopia. The information that you provide will be kept confidential by using only code numbers and locking the data.

Based on the understanding of the information I gave you, are you willing to participate in this study?

1. Yes
2. No
3. **Part 1:** **Health worker back ground information**
   1. Age
   2. Sex: □ Male □ Female
   3. Marital status: □ Single □ Married □ Divorce □ Widowed
   4. Type of health care setting:

□ Hospital □ Outpatient clinic □ Health center □ Home care for mild cases

□ Other: ________________

- 1. No of Children: □ No child □ 1-3 Children □ >3 Children
  2. Education level: □ high school □ Diploma □ BSc □ MSc
  3. Experience: □ 1-10 years □ 11-20 years □ >21 years
  4. Health care facility unit type in which the health worker works?

□ Outpatient

□ Emergency

□ Medical unit

□ Intensive care unit

□Cleaning services

□ Laboratory

□ Pharmacy

□ Isolation center

□ Other, specify:______________

- 1. Type of health work:

□ Medical doctor

□ Registered nurse (midwife)

□ Radiology /x-ray technician

□ Pharmacist/Pharmacy technician

□ public health expert

□ Laboratory personnel

□ Admission/reception clerk

□ Patient transporter

□ Driver

□ Cleaner

□ Other (specify): ______________

1. **Part 2:** **Assessment of exposure of health workers for COVID-19 (Exposed, probably exposed, Not exposed)**

| **S. No** | **Exposure assessment** | **Yes** | **No** | **Unknown** |
| --- | --- | --- | --- | --- |
| A | Did you provide direct care to a confirmed COVID-19 patient? |  |  |  |
| B | Did you have face-to-face contact (within 1 meter) with a confirmed COVID-19 patient in a health care facility? |  |  |  |
| C | Did you have direct contact with the environment where the confirmed COVID-19 patient was cared for? (bed, linen, medical equipment, bathroom etc.) |  |  |  |
| D | Were you present when any aerosol generating procedures (AGP) was performed on the patient? |  |  |  |

- 1. If yes for D, what type of aerosol generating procedure (AGP)?

□ Tracheal intubation

□ Nebulizer treatment

□ Open airway suctioning

□ Collection of sputum

□ Tracheostomy

□ Bronchoscopy

□ Cardiopulmonary resuscitation

□ Other, specify: ______________

- 1. Were you involved with health care interaction(s) (paid or unpaid) in another health care facility during the period above?

□ Other facility (public or private)

□ Ambulance

□ Home care

□ No other facility

- 1. If exposed, date of health worker first exposure to confirmed COVID-19 patient:

Date (DD/MM/YYYY): ___/___/______ □ Not known

1. **Part 3:** **Adherence to infection prevention and control (IPC) during health care interactions**
   1. During the period of a health care interaction with a COVID-19 patient, did you wear personal protective equipment (PPE)? □ Yes □ No
   2. If yes, for each item of PPE below, indicate how often you used it:

1. ‘Always, as recommended’ should be considered wearing the PPE when indicated more than 95% of the time;

2. ‘Most of the time’ should be considered 50% or more but not 95%

3. ‘occasionally’ should be considered 20% to under 50%

4. ‘Rarely’ should be considered less than 20%.

| S.No | Infection prevention and control (IPC) | 1 | 2 | 3 | 4 |
| --- | --- | --- | --- | --- | --- |
| 3.2.1 | Single gloves |  |  |  |  |
| 3.2.2 | Medical mask |  |  |  |  |
| 3.2.3 | Face shield or goggles/protective glasses |  |  |  |  |
| 3.2.4 | Disposable gown |  |  |  |  |
| 3.3. | did you perform hand hygiene before and after the following procedures | | | | |
| 3.3.1 | touching the COVID-19 patient |  |  |  |  |
| 3.3.2 | clean or aseptic procedure was performed |  |  |  |  |
| 3.3.3 | after exposure to body fluid |  |  |  |  |
| 3.3.4 | after touching the COVID-19 patient’s surroundings (bed, door handle, etc) |  |  |  |  |
| 3.4 | Did high touch surfaces decontaminated frequently (at least three times daily) |  |  |  |  |

3.5. did you remove and replace your medical mask if it became wet? □ Yes □ No

3.6. did you dispose the wet PPE in the waste bin? □ Yes □ No

3.7. did you perform hand hygiene procedure after you remove the PPE? □ Yes □ No

1. **Part 4:** **Adherence to infection prevention and control (IPC) during health care interactions (for those working on isolation centers)**
   1. During the period of Aerosol generating procedure (AGP) a health care interaction with a COVID-19 patient, did you wear personal protective equipment (PPE)? □ Yes □ No
   2. If yes, for each item of PPE below, indicate how often you used it:

1. ‘Always, as recommended’ should be considered wearing the PPE when indicated more than 95% of the time;

2. ‘Most of the time’ should be considered 50% or more but not 95%

3. ‘occasionally’ should be considered 20% to under 50%

4. ‘Rarely’ should be considered less than 20%.

| S.No | Infection prevention and control (IPC) | 1 | 2 | 3 | 4 |
| --- | --- | --- | --- | --- | --- |
| 4.2.1 | Single gloves |  |  |  |  |
| 4.2.2 | N95 mask |  |  |  |  |
| 4.2.3 | Face shield or goggles/protective glasses |  |  |  |  |
| 4.2.4 | Disposable gown |  |  |  |  |
| 4.2.5 | Water proof apron |  |  |  |  |
| 4.3. | did you perform hand hygiene before and after the following procedures | | | | |
| 4.3.1 | touching the COVID-19 patient |  |  |  |  |
| 4.3.2 | clean or aseptic procedure was performed |  |  |  |  |
| 4.3.3 | after exposure to body fluid |  |  |  |  |
| 4.3.4 | after touching the COVID-19 patient’s surroundings (bed, door handle, etc) |  |  |  |  |
| 4.4 | Did high touch surfaces decontaminated frequently (at least three times daily) |  |  |  |  |

4.5. did you remove and replace your medical mask if it became wet? □ Yes □ No

4.6. did you dispose the wet PPE in the waste bin? □ Yes □ No

4.7. did you perform hand hygiene procedure after you remove the PPE? □ Yes □ No

1. Did you have any episode of accidental exposure with biological fluid/respiratory secretions? □ Yes □ No
2. If yes, which type of accident?

□ Splash of biological fluid/respiratory secretions in the mucous membrane of eyes

□ Splash of biological ﬂuid/respiratory secreons in the mucous membrane of mouth/nose

□ Splash of biological fluid/respiratory secretions on non-intact skin

□ Puncture/sharp accident with any material contaminated with biological fluid/ respiratory secretions

1. Have you taken any training (in-service, online.) training regarding COVID-19? □ Yes □ No
