# Supplementary material for: Assessment of exposure risks to COVID-19 among frontline health care workers in Amhara Region, Ethiopia: A cross-sectional survey
Source: PLoS One. 2021 Apr 29;16(4):e0251000. doi: 10.1371/journal.pone.0251000 (PMC8084207; doi:10.1371/journal.pone.0251000)
Supplement: S2 File — (DOCX) [file pone.0251000.s002.docx]

**መጠይቅ 1፡ በጎንደር ዩኒቨርሲቲ በጤና ስራ ላይ ያለዉን የCOVID19የተጋላጭነት ሁኔታና ተያያዥ ምክኒያቶችን ለመለየት የሚደረግ የዳሰሳ ጥናት**

**የጥናት መረጃ፡** ይህ ጥናት በጎንደር ዩኒቨርሲቲ ተማራማሪዎች የሚካሄድ ሲሆን **በጎንደር ዩኒቨርሲቲ በጤና ስራ ላይ ያለዉን** የኮቨድ-19 **የተጋላጭነት ሁኔታና ተያያዥ ምክኒያቶችን ለመለየት የሚደረግ** የሚደረግ የዳሰሳ ጥናት ነዉ፡፡በዚህ ጥናት የሰጡት መረጃ የጤና ሰራተኞችየኮቨድ-19ያላቸዉን ተጋላጭነትና እያስከተለ ያለዉን የጤና ችግር ያሳያል፡፡ ጥናቱ ከመጀመሩ በፊት ህጋዊ ደብዳቤ ለ**ጎንደርዩኒቨርሲቲ ሆስፒታል ተልካል**፡፡

**የጥናቱ ጥቅም፡** በጥናቱ በመሳተፍዎ ምንም አይነት ቀጥተኛ ጥቅም ሊያገኙ አይችሉም፡፡ ነገር ግን የጥናቱ ግኝቶች **በጤና ስራ ላይ ያለዉን** የኮቨድ-19  **የተጋላጭነት ሁኔታና ተያያዥ ምክኒያቶችን ለመለየት ይረዳል**፡፡

**የጥናቱ ጉዳት:** በዚህ ጥናት ላይ መሳተፍ ለጥናቱ ቃለመጠይቅ ከሚፈጀዉ 15 ደቂቃወች በስተቀር ምንም አይነት ጉዳት ሊያደርስ አይችልም ፡፡

**የጥናቱ ተሳታፊወች መብት:** በዚህ ጥናት ላይ መሳተፍ በሙሉ ፈቃደኝነት ላይ የተመሰረተ ሲሆን፤ ማንኛዉም በጥናቱ ላይ ለመሳተፍ የተመረጠ ሰዉ በጥናቱ ያለመሳተፍ መብት አለዉ፡፡ የጥናቱ ተሳታፊወች በማንኛዉም በፈለጉበት ሰዓት ቃለመጠይቁን ማቋረጥ ይችላሉ፡፡ የጥናቱ ተሳታፊወች የመረጡትን ጥያቄወች ብቻ የመመለስና ያልፈለጉትን ያለመመለስ መብት አላቸዉ፡፡ ማንኛዉም ሃሳብ ከቃለመጠይቁ በፊት፤ መሃል ወይም በኃላ ቢነሳ መረጃ ሰብሳቢዎች ማስረዳት ይገባቸዋል፡፡

**ሚስጥራዊነት፡** ማንኛዉም በዚህ ጥናት የተሰበሰበ መረጃ ለምርምር አገልግሎት ብቻ ይዉላል፡፡ ሚስጥራዊነትን ለማስጠበቅ የጥናቱን ተሳታፊወች በመለያ ቁጥሮች ተክተን የምንጠቀም ሲሆን መለያ ስሞችን አንጠቀምም፡፡

**የዋና ተመራማሪ አድራሻ፡**በጥናቱ ዙሪያ ተጨማሪ መረጃ የሚስፈልጋችሁ ከሆነ በሚከተለዉ አድራሻ መጠየቅ ትችላላችሁ፡፡ (ሞባይል = 0920534469, ኢሜል = seyepharma@gmail.com)

**ፈቃድ መጠየቂያ ቅፅ**

ስሜ-----------------------ይባላል፡፡ በጎንደር ዩኒቨርሲቲ በህክምናና ጤና ሳይንስ ኮሌጅ ስር ከሚካሄዱ የምርምር ስራወች ዉስጥ **በጤና ስራ ላይ ያለዉን** የኮቨድ-19 **የተጋላጭነት ሁኔታና ተያያዥ ምክኒያቶችን ለመለየት** ከሚያጠናዉ የጥናት ቡድን አንዱ አባል ነኝ፡፡ በአጠቃለይ የጥናቱ ሁኔታና ከእርስዎ የሚጠበቀዉ ነገር ግልፅ ከሆነልዎ በጥናቱ ዉስጥ ለመሳተፍ ስለተመረጡ በታማኝነት ቢሳተፉ የጥናቱን ዓላማ ከማሳካት አንፃር ትልቅ አስተዋፅዖ አለዉ፡፡ሰለዚህ የእርስዎን ትክክለኛ መልስ በአክብሮት እንጠይቃለን፡፡ በጥናቱ ዉስጥ ለመሳተፍ ፈቃደኛ ነዎት?

አወ፤ በጥናቱ ዉስጥ ለመሳተፍ እፈልጋለሁ (እባክዎትን ወደሚቀጥለዉ ገፅ ይቀጥሉ)

የለም፤ በጥናቱ ዉስጥ መሳተፍ አልፈልግም (ያቁሙ)

በጣም አመሰግናለሁ!!

የመጠይቁ መለያ ቁጥር __________

የመረጃ ሰብሳቢው ስም _____________________ፊርማ _____________ ቀን_____________

የተቆጣጣሪው ስም________________________ፊርማ _____________ ቀን_____________

1. የጤና ባለሙያዉ ግላዊ መረጃ

1.1. ዕድሜ--------------------ዓመት

1.2. ፆታ: - □ ወንድ □ ሴት

1.3 የጋብቻ ሁኔታ □ ያላገባ □ ያገባ □ የተፋታ □ በሞት የተለየ

1.4 ሙያ □ ሀኪም □ ነርስ □ ላብራቶሪ □ ፋርማሲ □ሌላ ---------------------

1.5. የጤና እንክብካቤ ክል: □ ሆስፒታል □ የተመላላሽ ክሊኒክ □ የጤና ማእከል ለአነስተኛ ጉዳዮች የቤት

ውስጥ እንክብካቤ ሌላ ________________

1.6 በቤትዎ ዉስጥ ያሉት የልጆች ቁጥር □ልጅ የለም □ 1-3 ልጆች አሉ □> 3 ልጆች አሉ

1.7 የትምህርት ደረጃ □ዲፕሎማ □ የመጀመሪያ ድግሪ □ሁለተኛ ድግሪ □ሶስተኛ ድግሪ

1.8 የስራ ልምድ □1-10 ዓመት □11-20 ዓመት □ከ21 ዓመት በላይ

1.9 የህክምና አገልግሎት የሚሰጡበት ክፍል □ተመላላሽ ህክምና □ድንገተኛ ህክምና □ተኝቶ ህክምና

□ፅኑ ህሙማን ህክምና □ፅዳት አገልግሎት □የናሙና ምርመራ ክፍል □መድኃኒት ቤት

□በለይቶ ማቆያ ክፍል □ሌላ --------------

1.10. የጤና ሥራ ዓይነት □ የሕክምና ዶክተር □ የተመዘገበ ነርስ (አዋላጅ) □ የታካሚ አጓጓዥ

□ የራዲዮሎጂ/ኤክስሬይ ቴክኒሽያን □ ፋርማሲስት/የመድኃኒት ባለሙያ □ ህሙማን ተቀባይ

□ የህዝብ ጤና ባለሙያ (ጤና መኮንን) □ የላቦራቶሪ ባለሙያ □ፅዳት □ሌላ------------

2. የጤና ሰራተኞች ለኮቨድ-19 ያላቸዉ ተጋላጭነት (የተጋለጠ ፣ በከፊል የተጋለጠ ፣ ምንም ያልተጋለጠ)

| ተ.ቁ | የተጋላጭነት ግምገማ | አዎ | የለም | አይታወቅም |
| --- | --- | --- | --- | --- |
| ሀ | ለተረጋገጠ የኮቨድ-19 ህመምተኛ ቀጥተኛ እንክብካቤን ሰጥተዋልን? |  |  |  |
| ለ | ለተረጋገጠ የኮቨድ-19 ህመምተኛ ጋር ፊት ለፊት (በ1 ሜትር) ተገናኝተዋል? |  |  |  |
| ሐ | ለተረጋገጠ የኮቨድ-19 ህመምተኛ እንክብካቤ ከተደረገበት አካባቢ ጋር ቀጥተኛ ግንኙነት አልዎት? (አልጋ ፣ ጨርቃ ጨርቅ ፣ የህክምና መሳሪያ ፣ መታጠቢያ ቤት ወዘተ) |  |  |  |
| መ | ለኮቨድ-19 ህመምተኞች የአየር ማራዘሚያ ሂደት ስራ ሲሰራ በአካባቢዉ ነበሩ? |  |  |  |

2.1. ለጥያቄ ፤መ፣ መልስዎ አዎ ከሆነ ለምን ዓይነት የአየር ማራዘሚያ ሂደት (AGP)?

ሀ. የአየር ቱቦ ሰንጥቆ መተንሻ ሲዘጋጅ ለ. የመድሃኒት ቅንጣቶችን ወደ መተንፈሻ አካላት ሲሪጭ

ሐ. የአየር መተንፈሻ ሲመጠጥ መ.የአክታ ናሙና ሲሰበሰብ

ሰ. የልብ ምትንና መተንፈሻ አካልን በሰው ሠራሽ አተነፋፈስ ለመመለስ ሲሳራ ሪ. የአየር ቱቦን ውስጠኛ ክፍል

ሲመሪመር ሠ.የአየር ቱቦ ቆርጦ ሲቀጠል ሸ. ሌላ ፣ ይግለጹ ______________

2.2. ከላይ በተጠቀሰው ጊዜ በሌላ የጤና ተቋም ውስጥ ከጤና እንክብካቤ ጋር ግንኙነት ነበርዎ? □ አዎ

□ የለም አዎ ካሉ፡- □ ሌላ ተቋም (የህዝብ ወይስ የግል) □ የቤት ውስጥ እንክብካቤ

□ አምቡላንስ □ ሌላ ፣ ይግለጹ ______________

2.3 . የኮቨድ-19 ህመምተኛ ጋር ንክኪ ያለዎ ( የተጋለጡ) ነዎት? መልስዎ አዎ ከሆነ ቀኑን ይጥቀሱ------------

------------/ዓ.ም. ለ. ቀኑ አይታወቅም

3. የኮቨድ-19 በጤና እንክብካቤ ግንኙነቶች ወቅት የኢንፌክሽን መከላከል እና ቁጥጥርን (IPC) ስለመከተል፡- 3.1. የኮቨድ-19 ህመምተኛ የጤና እንክብካቤ በሚደግበት ወቅት የግል መከላከያ መሳሪያ (PPE) ለብሰዋል?

□ አዎ □ የለም

3.2. ለጥያቄ 3.1 መልስዎ አዎ ከሆነ ከታች ለተዘዙት የግል መከላከያ መሳሪያዎች ምን ያህል ጊዜ እንደሚጠቀሙ ያመልክቱ-

1. 'ሁል ጊዜ' እንደሚመከረው ከ 95% በላይ ጊዜየን የግል መከላከያ መሳሪያዎችን እለብሳለሁ፣

2. 'አብዛኛው ጊዜ' 50% ወይም ከዚያ በላይ ነገ ግን ከ95% በታች የግል መከላከያ መሳሪያዎችን እለብሳለሁ፣

3. 'አልፎ አልፎ' ከ20%-50% የግል መከላከያ መሳሪያዎችን እለብሳለሁ፣

4. 'በጣም ለጥቂት ጊዜ' ከ20% በታች የግል መከላከያ መሳሪያዎችን እለብሳለሁ፣

| ተ.ቁ | የኢንፌክሽን መከላከል እና ቁጥጥር (IPC) | 1 | 2 | 3 | 4 |
| --- | --- | --- | --- | --- | --- |
| 3.2.1 | ¹ንት |  |  |  |  |
| 3.2.2 | የህክምና ጭምብል |  |  |  |  |
| 3.2.3 | የፈት መከላከያ ልብስ ወይም የመከላከያ መነፅሮች |  |  |  |  |
| 3.2.4 | አንዴ ብቻ የሚያገለግል ጋዉን |  |  |  |  |
| 3.3 | ከሚከተሉት ተግባራት በትና በኃላ የእጅ ንፅህናን(መታጠብ) ያከናዉናሉ |  |  |  |  |
| 3.3.1 | የኮቨድ-19 ህመምተኛ ጋ ንክኪ በሚያደጉበት ጊዜ |  |  |  |  |
| 3.3.2 | የፅዳት ስራ ወይም የቁስልን ንክኪ በሚያጋጥምበት ጊዜ |  |  |  |  |
| 3.3.3 | ከሰዉነት በሚወጡ ሳሾች ጋ ንክኪ በሚያጋጥምበት ጊዜ |  |  |  |  |
| 3.3.4 | የኮቨድ-19 ህመምተኛ የሚጠቀምባቸዉን(አልጋ፣የበ እጀታ፣ ወዘተ) በነካሁበት ጊዜ |  |  |  |  |
| 3. 4 | ብዙ ጊዜ ለንክኪ የተጋለጡ ወለሎችን ሲያፀዱ ( ቢያንስ በቀን 3 ጊዜ) |  |  |  |  |

3.5 የሚጠቀሙት የህክምና ጭምብል እርጥበት በሚያገኝበት ጊዜ ያስወግዱታል ወይስ በሌላ ይተኩታል

□ አዎ □የለም

3.6 እርጥቡን የህክምና ጭምብል በቆሻሻ ማጠራቀሚያ ዉስጥ ያስወግዳሉ □ አዎ □ የለም

3.7 የህክምና ጭምብሉን ካስወገዱ በኃላ እጅዎን ይታጠባሉ □ አዎ □ የለም

4. በጤና እንክብካቤ ወቅት በሽታን የመከላከልና የመቆጣጠር (IPC) ሂደትን ይከተላሉ (በለይቶ ማቆያ ለሚሰራ)፡፡

4.1. የኮቨድ-19 ህመምተኛ የአየር ማራዘሚያ (ማሰራጫ) ሂደት በሚሰጥበት ጊዜ የግል መከላከያ መሳያዎችን

(PPE) ለብሰዋል? □ አዎ □ የለም

4.2. ለጥያቄ 4.1 መልስዎ አዎ ከሆነ ከታች የተዘዘት የግል መከላከያ መሳያዎችን ምን ያህል ጊዜ እንደሚጠቀሙ ያመልክቱ-

1. 'ሁል ጊዜ' እንደሚመከረው ከ 95% በላይ ጊዜየን የግል መከላከያ መሳሪያዎችን እለብሳለሁ፣

2. 'አብዛኛው ጊዜ' 50% ወይም ከዚያ በላይ ነገ ግን ከ95% በታች የግል መከላከያ መሳሪያዎችን እለብሳለሁ፣

3. 'አልፎ አልፎ' ከ20%-50% የግል መከላከያ መሳሪያዎችን እለብሳለሁ፣

4. 'በጣም ለጥቂት ጊዜ' ከ20% በታች የግል መከላከያ መሳሪያዎችን እለብሳለሁ፣

| ተ.ቁ | የኢንፌክሽን መከላከል እና ቁጥጥር (IPC) | 1 | 2 | 3 | 4 |
| --- | --- | --- | --- | --- | --- |
| 4.2.1 | ነጠላ ¹ንት |  |  |  |  |
| 4.2.2 | ኤን 95 ጭምብል |  |  |  |  |
| 4.2.3 | የፈት መከላከያ ልብስ ወይም የመከላከያ መነፅሮች |  |  |  |  |
| 4.2.4 | አንዴ ብቻ የሚያገለግል ጋዉን |  |  |  |  |
| 4.2.5 | ዉሃ የሚያሰርግ ልብስ( ሸታ) |  |  |  |  |
| 4.3 | ከሚከተሉት ተግባራት በትና በኃላ እጅዎን ይታጠባሉ |  |  |  |  |
| 4.3.1 | የኮቨድ-19 ህመምተኛ ጋ ንክኪ በሚያደጉበት ጊዜ |  |  |  |  |
| 4.3.2 | የፅዳት ስራ ወይም የቁስል ንክኪ በሚያጋጥምበት ጊዜ |  |  |  |  |
| 4.3.3 | ከሰዉነት በሚወጡ ሳሾች ጋ ንክኪ በሚያጋጥምበት ጊዜ |  |  |  |  |
| 4.3.4 | የኮቨድ-19 ህመምተኛ የሚጠቀምባቸዉን(አልጋ፣የበ እጀታ፣ ወዘተ) በነካሁበት ጊዜ |  |  |  |  |
| 4.4 | ብዙ ጊዜ ለንክኪ የተጋለጡ ወለሎችን ሲያፀዱ ( ቢያንስ በቀን 3 ጊዜ) |  |  |  |  |

4.5 የሚጠቀሙት የህክምና ጭምብል እርጥበት ሲኖዉ ያስወግዱታል ወይስ በሌላ ይተኩታል □አዎ □የለም

4.6 እርጥበት ያለዉን የህክምና ጭምብል በቆሻሻ ማጠራቀሚያ ዉስጥ ያስወግዳሉ □ አዎ □ የለም

4.7 የህክምና ጭምብሉን ካስወገዱ በኃላ እጅዎን ይታጠባሉ □ አዎ □ የለም

5. ከሰዉነት የሚወጡ ፈሳሾች / የመተንፈሻ አካላት ፈሳሽ/ በድንገት የመጋለጥ አጋጣሚ አልዎት? □ አዎ □የለም

6. ለጥያቄ ቁጥ 5 መልስዎ አዎ ከሆነ ፣ የትኛው አደጋ ነው?

□ ከሰዉነት/ ከመተንፈሻ አካል የወጣ ፍሳሽ ዓይኔን መረጨት

□ ከሰዉነት/ ከመተንፈሻ አካል የወጣ ፍሳሽ በአፍ / በአፍንጫ ውስጥ መረጨት

□ ከሰዉነት/ ከመተንፈሻ አካል የወጣ ፍሳሽ ቆዳዬ ላይ መፍሰስ

□ ከሰዉነት/ ከመተንፈሻ አካል በወጣ ፍሳሽ የተበከለ ስለታም ነገር መወጋት

7. በስራ ላይ ሆነዉ ወይም በቀጥታ በኢንተኔት ስለኮቨድ-19 ስልጠና ወስደዋል □ አዎ □የለም

***ለነበዎት ቆይታ እናመሰግናለን!!***
