# Supplementary material for: Assessment of exposure risks to COVID-19 among frontline health care workers in Amhara Region, Ethiopia: A cross-sectional survey
Source: PLoS One. 2021 Apr 29;16(4):e0251000. doi: 10.1371/journal.pone.0251000 (PMC8084207; doi:10.1371/journal.pone.0251000)
Supplement: S3 File — (DOCX) [file pone.0251000.s003.docx]

| **Case Processing Summary** | | | |
| --- | --- | --- | --- |
| Unweighted Cases^a^ | | N | Percent |
| Selected Cases | Included in Analysis | 418 | 100.0 |
|  | Missing Cases | 0 | .0 |
|  | Total | 418 | 100.0 |
| Unselected Cases | | 0 | .0 |
| Total | | 418 | 100.0 |
| a. If weight is in effect, see classification table for the total number of cases. | | | |

| **Classification Table^a,b^** | | | | | |
| --- | --- | --- | --- | --- | --- |
|  | Observed | | Predicted | | |
|  |  |  | Are you exposed to a confirmed COVID-19 patient? | | Percentage Correct |
|  |  |  | Yes | No |  |
| Step 0 | Are you exposed to a confirmed COVID-19 patient? | Yes | 0 | 78 | .0 |
|  |  | No | 0 | 340 | 100.0 |
|  | Overall Percentage | |  |  | 81.3 |
| a. Constant is included in the model. | | |  |  |  |
| b. The cut value is .500 | |  |  |  |  |

| **Classification Table^a^** | | | | | |
| --- | --- | --- | --- | --- | --- |
|  | Observed | | Predicted | | |
|  |  |  | Are you exposed to a confirmed COVID-19 patient? | | Percentage Correct |
|  |  |  | Yes | No |  |
| Step 1 | Are you exposed to a confirmed COVID-19 patient? | Yes | 0 | 78 | .0 |
|  |  | No | 0 | 340 | 100.0 |
|  | Overall Percentage | |  |  | 81.3 |
| a. The cut value is .500 | |  |  |  |  |

| **Variables in the Equation** | | | | | | | | | |
| --- | --- | --- | --- | --- | --- | --- | --- | --- | --- |
|  | | B | S.E. | Wald | df | Sig. | Exp(B) | 95.0% C.I.for EXP(B) | |
|  |  |  |  |  |  |  |  | Lower | Upper |
| Step 1^a^ | training | .756 | .258 | 8.595 | 1 | .003 | 2.130 | 1.285 | 3.532 |
|  | Constant | .232 | .429 | .292 | 1 | .589 | 1.261 |  |  |
| a. Variable(s) entered on step 1: training. | | | | |  |  |  |  |  |

| **Case Processing Summary** | | | |
| --- | --- | --- | --- |
| Unweighted Cases^a^ | | N | Percent |
| Selected Cases | Included in Analysis | 418 | 100.0 |
|  | Missing Cases | 0 | .0 |
|  | Total | 418 | 100.0 |
| Unselected Cases | | 0 | .0 |
| Total | | 418 | 100.0 |
| a. If weight is in effect, see classification table for the total number of cases. | | | |

| **Classification Table^a,b^** | | | | | |
| --- | --- | --- | --- | --- | --- |
|  | Observed | | Predicted | | |
|  |  |  | Are you exposed to a confirmed COVID-19 patient? | | Percentage Correct |
|  |  |  | Yes | No |  |
| Step 0 | Are you exposed to a confirmed COVID-19 patient? | Yes | 0 | 78 | .0 |
|  |  | No | 0 | 340 | 100.0 |
|  | Overall Percentage | |  |  | 81.3 |
| a.Constant is included in the model. | | |  |  |  |
| b.The cut value is .500 | |  |  |  |  |

| **Variables not in the Equation** | | | | | |
| --- | --- | --- | --- | --- | --- |
|  | | | Score | df | Sig. |
| Step 0 | Variables | Agegroup | 9.072 | 3 | .028 |
|  |  | Agegroup(1) | .346 | 1 | .556 |
|  |  | Agegroup(2) | 1.866 | 1 | .172 |
|  |  | Agegroup(3) | .682 | 1 | .409 |
|  |  | Maritalstatus | 5.997 | 2 | .050 |
|  |  | Maritalstatus(1) | 5.683 | 1 | .017 |
|  |  | Maritalstatus(2) | .032 | 1 | .857 |
|  |  | Typeofhealthcaresetting | 5.527 | 4 | .237 |
|  |  | Typeofhealthcaresetting(1) | .682 | 1 | .409 |
|  |  | Typeofhealthcaresetting(2) | .485 | 1 | .486 |
|  |  | Typeofhealthcaresetting(3) | .183 | 1 | .669 |
|  |  | Typeofhealthcaresetting(4) | 3.524 | 1 | .060 |
|  |  | family | 10.959 | 2 | .004 |
|  |  | family(1) | 7.769 | 1 | .005 |
|  |  | family(2) | .718 | 1 | .397 |
|  |  | Educationlevel | 3.163 | 4 | .531 |
|  |  | Educationlevel(1) | .652 | 1 | .419 |
|  |  | Educationlevel(2) | .930 | 1 | .335 |
|  |  | Educationlevel(3) | 1.752 | 1 | .186 |
|  |  | Educationlevel(4) | .183 | 1 | .669 |
|  |  | Experience | 73.026 | 3 | .000 |
|  |  | Experience(1) | 6.841 | 1 | .009 |
|  |  | Experience(2) | 2.207 | 1 | .137 |
|  |  | Experience(3) | 67.177 | 1 | .000 |
|  |  | Healthcarefacilityunittype | 38.984 | 6 | .000 |
|  |  | Healthcarefacilityunittype(1) | 23.017 | 1 | .000 |
|  |  | Healthcarefacilityunittype(2) | .390 | 1 | .532 |
|  |  | Healthcarefacilityunittype(3) | 2.649 | 1 | .104 |
|  |  | Healthcarefacilityunittype(4) | 15.179 | 1 | .000 |
|  |  | Healthcarefacilityunittype(5) | 1.266 | 1 | .261 |
|  |  | Healthcarefacilityunittype(6) | 4.478 | 1 | .034 |
|  |  | Typeofhealthwork | 54.498 | 6 | .000 |
|  |  | Typeofhealthwork(1) | .901 | 1 | .343 |
|  |  | Typeofhealthwork(2) | .000 | 1 | .993 |
|  |  | Typeofhealthwork(3) | 1.038 | 1 | .308 |
|  |  | Typeofhealthwork(4) | 18.079 | 1 | .000 |
|  |  | Typeofhealthwork(5) | 1.528 | 1 | .216 |
|  |  | Typeofhealthwork(6) | 3.630 | 1 | .057 |
|  |  | exposurestatus | 23.117 | 2 | .000 |
|  |  | exposurestatus(1) | 21.673 | 1 | .000 |
|  |  | exposurestatus(2) | .731 | 1 | .392 |
|  |  | PPEuse(1) | 1.118 | 1 | .290 |
|  |  | Handwashing(1) | 14.015 | 1 | .000 |
|  |  | hightouchsurfacesdecontaminatedfrequently(1) | 3.817 | 1 | .051 |
|  |  | wetmedicalmask(1) | 3.791 | 1 | .052 |
|  |  | PPEdisposal(1) | 4.472 | 1 | .034 |
|  |  | training(1) | 8.822 | 1 | .003 |
|  | Overall Statistics | | 152.463 | 38 | .000 |

| **Classification Table^a^** | | | | | |
| --- | --- | --- | --- | --- | --- |
|  | Observed | | Predicted | | |
|  |  |  | Are you exposed to a confirmed COVID-19 patient? | | Percentage Correct |
|  |  |  | Yes | No |  |
| Step 1 | Are you exposed to a confirmed COVID-19 patient? | Yes | 41 | 37 | 52.6 |
|  |  | No | 12 | 328 | 96.5 |
|  | Overall Percentage | |  |  | 88.3 |
| a. The cut value is .500 | |  |  |  |  |

| **Variables in the Equation** | | | | | | | | | |
| --- | --- | --- | --- | --- | --- | --- | --- | --- | --- |
|  | | B | S.E. | Wald | df | Sig. | Exp(B) | 95.0% C.I.for EXP(B) | |
|  |  |  |  |  |  |  |  | Lower | Upper |
| Step 1^a^ | Agegroup |  |  | 6.306 | 3 | .098 |  |  |  |
|  | Agegroup(1) | -1.936 | .909 | 4.531 | 1 | .033 | .144 | .024 | .858 |
|  | Agegroup(2) | -2.220 | 1.050 | 4.470 | 1 | .034 | .109 | .014 | .850 |
|  | Agegroup(3) | .075 | 1.600 | .002 | 1 | .963 | 1.078 | .047 | 24.819 |
|  | Maritalstatus |  |  | 4.130 | 2 | .127 |  |  |  |
|  | Maritalstatus(1) | .326 | .475 | .473 | 1 | .492 | 1.386 | .547 | 3.512 |
|  | Maritalstatus(2) | -2.047 | 1.206 | 2.879 | 1 | .090 | .129 | .012 | 1.374 |
|  | Typeofhealthcaresetting |  |  | 1.213 | 4 | .876 |  |  |  |
|  | Typeofhealthcaresetting(1) | .516 | 1.312 | .155 | 1 | .694 | 1.676 | .128 | 21.922 |
|  | Typeofhealthcaresetting(2) | .003 | .707 | .000 | 1 | .997 | 1.003 | .251 | 4.006 |
|  | Typeofhealthcaresetting(3) | .749 | 1.032 | .527 | 1 | .468 | 2.115 | .280 | 15.979 |
|  | Typeofhealthcaresetting(4) | 1.040 | 1.386 | .563 | 1 | .453 | 2.831 | .187 | 42.826 |
|  | family |  |  | 3.522 | 2 | .172 |  |  |  |
|  | family(1) | .537 | .446 | 1.451 | 1 | .228 | 1.712 | .714 | 4.104 |
|  | family(2) | 1.150 | .634 | 3.292 | 1 | .070 | 3.157 | .912 | 10.933 |
|  | Educationlevel |  |  | .659 | 4 | .956 |  |  |  |
|  | Educationlevel(1) | -.111 | .558 | .040 | 1 | .842 | .895 | .300 | 2.671 |
|  | Educationlevel(2) | -.194 | .741 | .068 | 1 | .794 | .824 | .193 | 3.523 |
|  | Educationlevel(3) | -.680 | .988 | .474 | 1 | .491 | .507 | .073 | 3.512 |
|  | Educationlevel(4) | .164 | 1.045 | .025 | 1 | .875 | 1.179 | .152 | 9.132 |
|  | Experience |  |  | 19.330 | 3 | .000 |  |  |  |
|  | Experience(1) | .708 | .631 | 1.259 | 1 | .262 | 2.029 | .590 | 6.982 |
|  | Experience(2) | -.589 | .841 | .490 | 1 | .484 | .555 | .107 | 2.886 |
|  | Experience(3) | -4.484 | 1.097 | 16.703 | 1 | .000 | .011 | .001 | .097 |
|  | Healthcarefacilityunittype |  |  | 8.245 | 6 | .221 |  |  |  |
|  | Healthcarefacilityunittype(1) | -.054 | .592 | .008 | 1 | .927 | .947 | .297 | 3.025 |
|  | Healthcarefacilityunittype(2) | .491 | .599 | .672 | 1 | .412 | 1.634 | .505 | 5.282 |
|  | Healthcarefacilityunittype(3) | -2.003 | 2.137 | .878 | 1 | .349 | .135 | .002 | 8.899 |
|  | Healthcarefacilityunittype(4) | 1.803 | 1.565 | 1.328 | 1 | .249 | 6.070 | .283 | 130.356 |
|  | Healthcarefacilityunittype(5) | 1.493 | 1.223 | 1.490 | 1 | .222 | 4.450 | .405 | 48.885 |
|  | Healthcarefacilityunittype(6) | 1.731 | .895 | 3.741 | 1 | .053 | 5.646 | .977 | 32.619 |
|  | Typeofhealthwork |  |  | 7.864 | 6 | .248 |  |  |  |
|  | Typeofhealthwork(1) | .800 | .590 | 1.842 | 1 | .175 | 2.226 | .701 | 7.068 |
|  | Typeofhealthwork(2) | -.073 | 1.006 | .005 | 1 | .942 | .930 | .129 | 6.672 |
|  | Typeofhealthwork(3) | 1.402 | 1.012 | 1.919 | 1 | .166 | 4.063 | .559 | 29.522 |
|  | Typeofhealthwork(4) | 2.178 | 1.359 | 2.566 | 1 | .109 | 8.825 | .615 | 126.691 |
|  | Typeofhealthwork(5) | 2.050 | 2.218 | .854 | 1 | .355 | 7.765 | .101 | 599.423 |
|  | Typeofhealthwork(6) | 2.026 | .927 | 4.774 | 1 | .029 | 7.587 | 1.232 | 46.722 |
|  | exposurestatus |  |  | 20.173 | 2 | .000 |  |  |  |
|  | exposurestatus(1) | 2.317 | .516 | 20.125 | 1 | .000 | 10.145 | 3.687 | 27.918 |
|  | exposurestatus(2) | .881 | .557 | 2.506 | 1 | .113 | 2.415 | .811 | 7.191 |
|  | PPEuse(1) | .248 | .405 | .376 | 1 | .540 | 1.282 | .579 | 2.838 |
|  | Handwashing(1) | -.702 | .405 | 2.998 | 1 | .083 | .496 | .224 | 1.097 |
|  | hightouchsurfacesdecontaminatedfrequently(1) | .780 | .419 | 3.460 | 1 | .063 | 2.182 | .959 | 4.964 |
|  | wetmedicalmask(1) | -.365 | .533 | .469 | 1 | .493 | .694 | .244 | 1.972 |
|  | PPEdisposal(1) | -1.122 | .650 | 2.982 | 1 | .084 | .326 | .091 | 1.164 |
|  | training(1) | .055 | .462 | .014 | 1 | .906 | 1.056 | .427 | 2.612 |
|  | Constant | 2.515 | 1.226 | 4.211 | 1 | .040 | 12.368 |  |  |
| a. Variable(s) entered on step 1: Agegroup, Maritalstatus, Typeofhealthcaresetting, family, Educationlevel, Experience, Healthcarefacilityunittype, Typeofhealthwork, exposurestatus, PPEuse, Handwashing, hightouchsurfacesdecontaminatedfrequently, wetmedicalmask, PPEdisposal, training. | | | | | | | | | |

| **Case Processing Summary** | | | |
| --- | --- | --- | --- |
| Unweighted Cases^a^ | | N | Percent |
| Selected Cases | Included in Analysis | 418 | 100.0 |
|  | Missing Cases | 0 | .0 |
|  | Total | 418 | 100.0 |
| Unselected Cases | | 0 | .0 |
| Total | | 418 | 100.0 |
| a. If weight is in effect, see classification table for the total number of cases. | | | |

| **Classification Table^a,b^** | | | | | |
| --- | --- | --- | --- | --- | --- |
|  | Observed | | Predicted | | |
|  |  |  | Are you exposed to a confirmed COVID-19 patient? | | Percentage Correct |
|  |  |  | Yes | No |  |
| Step 0 | Are you exposed to a confirmed COVID-19 patient? | Yes | 0 | 78 | .0 |
|  |  | No | 0 | 340 | 100.0 |
|  | Overall Percentage | |  |  | 81.3 |
| a. Constant is included in the model. | | |  |  |  |
| b. The cut value is .500 | |  |  |  |  |

| **Variables not in the Equation** | | | | | |
| --- | --- | --- | --- | --- | --- |
|  | | | Score | df | Sig. |
| Step 0 | Variables | Agegroup | 9.072 | 3 | .028 |
|  |  | Agegroup(1) | .346 | 1 | .556 |
|  |  | Agegroup(2) | 1.866 | 1 | .172 |
|  |  | Agegroup(3) | .682 | 1 | .409 |
|  | Overall Statistics | | 9.072 | 3 | .028 |

| **Variables in the Equation** | | | | | | | | | |
| --- | --- | --- | --- | --- | --- | --- | --- | --- | --- |
|  | | B | S.E. | Wald | df | Sig. | Exp(B) | 95.0% C.I.for EXP(B) | |
|  |  |  |  |  |  |  |  | Lower | Upper |
| Step 1^a^ | Agegroup |  |  | 8.624 | 3 | .035 |  |  |  |
|  | Agegroup(1) | .801 | .334 | 5.771 | 1 | .016 | 2.229 | 1.159 | 4.285 |
|  | Agegroup(2) | 1.192 | .464 | 6.606 | 1 | .010 | 3.292 | 1.327 | 8.167 |
|  | Agegroup(3) | 1.580 | 1.090 | 2.104 | 1 | .147 | 4.857 | .574 | 41.107 |
|  | Constant | .722 | .296 | 5.967 | 1 | .015 | 2.059 |  |  |
| a. Variable(s) entered on step 1: Agegroup. | | | | |  |  |  |  |  |

| **sum of PPE variables** | | | | | |
| --- | --- | --- | --- | --- | --- |
|  | | Frequency | Percent | Valid Percent | Cumulative Percent |
| Valid | 5 | 25 | 6.0 | 6.0 | 6.0 |
|  | 6 | 6 | 1.4 | 1.4 | 7.4 |
|  | 7 | 16 | 3.8 | 3.8 | 11.2 |
|  | 8 | 9 | 2.2 | 2.2 | 13.4 |
|  | 9 | 36 | 8.6 | 8.6 | 22.0 |
|  | 10 | 15 | 3.6 | 3.6 | 25.6 |
|  | 11 | 47 | 11.2 | 11.2 | 36.8 |
|  | 12 | 26 | 6.2 | 6.2 | 43.1 |
|  | 13 | 49 | 11.7 | 11.7 | 54.8 |
|  | 14 | 52 | 12.4 | 12.4 | 67.2 |
|  | 15 | 41 | 9.8 | 9.8 | 77.0 |
|  | 16 | 15 | 3.6 | 3.6 | 80.6 |
|  | 17 | 39 | 9.3 | 9.3 | 90.0 |
|  | 18 | 13 | 3.1 | 3.1 | 93.1 |
|  | 19 | 8 | 1.9 | 1.9 | 95.0 |
|  | 20 | 2 | .5 | .5 | 95.5 |
|  | 21 | 12 | 2.9 | 2.9 | 98.3 |
|  | 22 | 6 | 1.4 | 1.4 | 99.8 |
|  | 25 | 1 | .2 | .2 | 100.0 |
|  | Total | 418 | 100.0 | 100.0 |  |

| **sum of hand washing variables** | | | | | |
| --- | --- | --- | --- | --- | --- |
|  | | Frequency | Percent | Valid Percent | Cumulative Percent |
| Valid | 5 | 108 | 25.8 | 25.8 | 25.8 |
|  | 6 | 12 | 2.9 | 2.9 | 28.7 |
|  | 7 | 14 | 3.3 | 3.3 | 32.1 |
|  | 8 | 21 | 5.0 | 5.0 | 37.1 |
|  | 9 | 49 | 11.7 | 11.7 | 48.8 |
|  | 10 | 31 | 7.4 | 7.4 | 56.2 |
|  | 11 | 21 | 5.0 | 5.0 | 61.2 |
|  | 12 | 18 | 4.3 | 4.3 | 65.6 |
|  | 13 | 29 | 6.9 | 6.9 | 72.5 |
|  | 14 | 14 | 3.3 | 3.3 | 75.8 |
|  | 15 | 2 | .5 | .5 | 76.3 |
|  | 16 | 7 | 1.7 | 1.7 | 78.0 |
|  | 17 | 33 | 7.9 | 7.9 | 85.9 |
|  | 18 | 5 | 1.2 | 1.2 | 87.1 |
|  | 19 | 5 | 1.2 | 1.2 | 88.3 |
|  | 20 | 6 | 1.4 | 1.4 | 89.7 |
|  | 21 | 37 | 8.9 | 8.9 | 98.6 |
|  | 22 | 2 | .5 | .5 | 99.0 |
|  | 24 | 1 | .2 | .2 | 99.3 |
|  | 25 | 3 | .7 | .7 | 100.0 |
|  | Total | 418 | 100.0 | 100.0 |  |

| **Descriptive Statistics** | | | | | |
| --- | --- | --- | --- | --- | --- |
|  | N | Minimum | Maximum | Mean | Std. Deviation |
| sum of hand washing variables | 418 | 5.00 | 25.00 | 10.9498 | 5.41601 |
| sum of PPE variables | 418 | 5.00 | 25.00 | 12.8373 | 4.00627 |
| Valid N (listwise) | 418 |  |  |  |  |

| **Variables in the Equation** | | | | | | | | | |
| --- | --- | --- | --- | --- | --- | --- | --- | --- | --- |
|  | | B | S.E. | Wald | df | Sig. | Exp(B) | 95.0% C.I.for EXP(B) | |
|  |  |  |  |  |  |  |  | Lower | Upper |
| Step 1^a^ | PPEuse(1) | .168 | .256 | .430 | 1 | .512 | 1.183 | .716 | 1.953 |
|  | Constant | 1.402 | .163 | 74.153 | 1 | .000 | 4.064 |  |  |
| a. Variable(s) entered on step 1: PPEuse. | | | | |  |  |  |  |  |

| **Variables in the Equation** | | | | | | | | | |
| --- | --- | --- | --- | --- | --- | --- | --- | --- | --- |
|  | | B | S.E. | Wald | df | Sig. | Exp(B) | 95.0% C.I.for EXP(B) | |
|  |  |  |  |  |  |  |  | Lower | Upper |
| Step 1^a^ | Handwashing(1) | -.528 | .264 | 4.010 | 1 | .045 | .590 | .352 | .989 |
|  | Constant | 1.792 | .212 | 71.546 | 1 | .000 | 6.000 |  |  |
| a. Variable(s) entered on step 1: Handwashing. | | | | |  |  |  |  |  |

| **Classification Table^a^** | | | | | |
| --- | --- | --- | --- | --- | --- |
|  | Observed | | Predicted | | |
|  |  |  | Are you exposed to a confirmed COVID-19 patient? | | Percentage Correct |
|  |  |  | Yes | No |  |
| Step 1 | Are you exposed to a confirmed COVID-19 patient? | Yes | 37 | 41 | 47.4 |
|  |  | No | 12 | 328 | 96.5 |
|  | Overall Percentage | |  |  | 87.3 |
| a. The cut value is .500 | |  |  |  |  |

| **Variables in the Equation** | | | | | | | | | |
| --- | --- | --- | --- | --- | --- | --- | --- | --- | --- |
|  | | B | S.E. | Wald | df | Sig. | Exp(B) | 95.0% C.I.for EXP(B) | |
|  |  |  |  |  |  |  |  | Lower | Upper |
| Step 1^a^ | Agegroup |  |  | 6.121 | 3 | .106 |  |  |  |
|  | Agegroup(1) | -1.621 | .806 | 4.045 | 1 | .044 | .198 | .041 | .960 |
|  | Agegroup(2) | -2.050 | .969 | 4.480 | 1 | .034 | .129 | .019 | .859 |
|  | Agegroup(3) | .220 | 1.572 | .020 | 1 | .889 | 1.246 | .057 | 27.120 |
|  | Maritalstatus |  |  | 3.583 | 2 | .167 |  |  |  |
|  | Maritalstatus(1) | .433 | .473 | .837 | 1 | .360 | 1.542 | .610 | 3.899 |
|  | Maritalstatus(2) | -1.638 | 1.152 | 2.022 | 1 | .155 | .194 | .020 | 1.858 |
|  | Typeofhealthcaresetting |  |  | 1.579 | 4 | .813 |  |  |  |
|  | Typeofhealthcaresetting(1) | .669 | 1.306 | .262 | 1 | .608 | 1.953 | .151 | 25.257 |
|  | Typeofhealthcaresetting(2) | -.166 | .693 | .057 | 1 | .811 | .847 | .218 | 3.292 |
|  | Typeofhealthcaresetting(3) | .776 | 1.052 | .544 | 1 | .461 | 2.173 | .276 | 17.086 |
|  | Typeofhealthcaresetting(4) | 1.261 | 1.441 | .766 | 1 | .382 | 3.530 | .209 | 59.499 |
|  | family |  |  | 4.507 | 2 | .105 |  |  |  |
|  | family(1) | .569 | .445 | 1.635 | 1 | .201 | 1.767 | .738 | 4.231 |
|  | family(2) | 1.328 | .641 | 4.294 | 1 | .038 | 3.774 | 1.075 | 13.256 |
|  | Educationlevel |  |  | 1.397 | 4 | .845 |  |  |  |
|  | Educationlevel(1) | -.116 | .554 | .044 | 1 | .834 | .890 | .301 | 2.637 |
|  | Educationlevel(2) | -.122 | .744 | .027 | 1 | .869 | .885 | .206 | 3.803 |
|  | Educationlevel(3) | -.851 | .991 | .738 | 1 | .390 | .427 | .061 | 2.976 |
|  | Educationlevel(4) | .551 | 1.080 | .261 | 1 | .610 | 1.736 | .209 | 14.407 |
|  | Experience |  |  | 24.601 | 3 | .000 |  |  |  |
|  | Experience(1) | 4.782 | 1.018 | 22.076 | 1 | .000 | 119.332 | 16.235 | 877.133 |
|  | Experience(2) | 5.386 | 1.156 | 21.712 | 1 | .000 | 218.250 | 22.653 | 2102.774 |
|  | Experience(3) | 3.976 | 1.272 | 9.772 | 1 | .002 | 53.310 | 4.407 | 644.924 |
|  | Healthcarefacilityunittype |  |  | 7.090 | 6 | .313 |  |  |  |
|  | Healthcarefacilityunittype(1) | -.215 | .580 | .137 | 1 | .711 | .807 | .259 | 2.515 |
|  | Healthcarefacilityunittype(2) | .325 | .582 | .311 | 1 | .577 | 1.384 | .442 | 4.331 |
|  | Healthcarefacilityunittype(3) | -2.014 | 2.331 | .746 | 1 | .388 | .134 | .001 | 12.868 |
|  | Healthcarefacilityunittype(4) | .921 | 1.480 | .387 | 1 | .534 | 2.512 | .138 | 45.667 |
|  | Healthcarefacilityunittype(5) | 1.231 | 1.237 | .991 | 1 | .320 | 3.424 | .303 | 38.648 |
|  | Healthcarefacilityunittype(6) | 1.618 | .903 | 3.210 | 1 | .073 | 5.042 | .859 | 29.590 |
|  | Typeofhealthwork |  |  | 9.306 | 6 | .157 |  |  |  |
|  | Typeofhealthwork(1) | .887 | .586 | 2.291 | 1 | .130 | 2.429 | .770 | 7.663 |
|  | Typeofhealthwork(2) | -.268 | 1.002 | .072 | 1 | .789 | .765 | .107 | 5.448 |
|  | Typeofhealthwork(3) | 1.483 | 1.017 | 2.127 | 1 | .145 | 4.406 | .600 | 32.321 |
|  | Typeofhealthwork(4) | 2.651 | 1.384 | 3.669 | 1 | .055 | 14.174 | .940 | 213.668 |
|  | Typeofhealthwork(5) | 1.940 | 2.400 | .654 | 1 | .419 | 6.961 | .063 | 768.153 |
|  | Typeofhealthwork(6) | 2.039 | .938 | 4.732 | 1 | .030 | 7.686 | 1.224 | 48.274 |
|  | exposurestatus |  |  | 19.471 | 2 | .000 |  |  |  |
|  | exposurestatus(1) | 2.257 | .512 | 19.459 | 1 | .000 | 9.558 | 3.506 | 26.058 |
|  | exposurestatus(2) | 1.011 | .545 | 3.448 | 1 | .063 | 2.749 | .945 | 7.993 |
|  | PPEuse(1) | .031 | .402 | .006 | 1 | .938 | 1.032 | .470 | 2.267 |
|  | Handwashing(1) | -.822 | .392 | 4.405 | 1 | .036 | .440 | .204 | .947 |
|  | hightouchsurfacesdecontaminatedfrequently(1) | .924 | .413 | 5.012 | 1 | .025 | 2.520 | 1.122 | 5.658 |
|  | wetmedicalmask(1) | -.670 | .513 | 1.709 | 1 | .191 | .512 | .187 | 1.397 |
|  | training(1) | .077 | .460 | .028 | 1 | .868 | 1.080 | .438 | 2.660 |
|  | Constant | -3.097 | 1.125 | 7.583 | 1 | .006 | .045 |  |  |
| a. Variable(s) entered on step 1: Agegroup, Maritalstatus, Typeofhealthcaresetting, family, Educationlevel, Experience, Healthcarefacilityunittype, Typeofhealthwork, exposurestatus, PPEuse, Handwashing, hightouchsurfacesdecontaminatedfrequently, wetmedicalmask, training. | | | | | | | | | |

| **Variables in the Equation** | | | | | | | | | |
| --- | --- | --- | --- | --- | --- | --- | --- | --- | --- |
|  | | B | S.E. | Wald | df | Sig. | Exp(B) | 95.0% C.I.for EXP(B) | |
|  |  |  |  |  |  |  |  | Lower | Upper |
| Step 1^a^ | Agegroup |  |  | 6.121 | 3 | .106 |  |  |  |
|  | Agegroup(1) | -1.621 | .806 | 4.045 | 1 | .044 | .198 | .041 | .960 |
|  | Agegroup(2) | -2.050 | .969 | 4.480 | 1 | .034 | .129 | .019 | .859 |
|  | Agegroup(3) | .220 | 1.572 | .020 | 1 | .889 | 1.246 | .057 | 27.120 |
|  | Maritalstatus |  |  | 3.583 | 2 | .167 |  |  |  |
|  | Maritalstatus(1) | .433 | .473 | .837 | 1 | .360 | 1.542 | .610 | 3.899 |
|  | Maritalstatus(2) | -1.638 | 1.152 | 2.022 | 1 | .155 | .194 | .020 | 1.858 |
|  | Typeofhealthcaresetting |  |  | 1.579 | 4 | .813 |  |  |  |
|  | Typeofhealthcaresetting(1) | .669 | 1.306 | .262 | 1 | .608 | 1.953 | .151 | 25.257 |
|  | Typeofhealthcaresetting(2) | -.166 | .693 | .057 | 1 | .811 | .847 | .218 | 3.292 |
|  | Typeofhealthcaresetting(3) | .776 | 1.052 | .544 | 1 | .461 | 2.173 | .276 | 17.086 |
|  | Typeofhealthcaresetting(4) | 1.261 | 1.441 | .766 | 1 | .382 | 3.530 | .209 | 59.499 |
|  | family |  |  | 4.507 | 2 | .105 |  |  |  |
|  | family(1) | .569 | .445 | 1.635 | 1 | .201 | 1.767 | .738 | 4.231 |
|  | family(2) | 1.328 | .641 | 4.294 | 1 | .038 | 3.774 | 1.075 | 13.256 |
|  | Educationlevel |  |  | 1.397 | 4 | .845 |  |  |  |
|  | Educationlevel(1) | -.116 | .554 | .044 | 1 | .834 | .890 | .301 | 2.637 |
|  | Educationlevel(2) | -.122 | .744 | .027 | 1 | .869 | .885 | .206 | 3.803 |
|  | Educationlevel(3) | -.851 | .991 | .738 | 1 | .390 | .427 | .061 | 2.976 |
|  | Educationlevel(4) | .551 | 1.080 | .261 | 1 | .610 | 1.736 | .209 | 14.407 |
|  | Experience |  |  | 24.601 | 3 | .000 |  |  |  |
|  | Experience(1) | .604 | .645 | .875 | 1 | .350 | 1.829 | .516 | 6.481 |
|  | Experience(2) | -.806 | .824 | .956 | 1 | .328 | .447 | .089 | 2.247 |
|  | Experience(3) | -4.782 | 1.018 | 22.076 | 1 | .000 | .008 | .001 | .062 |
|  | Healthcarefacilityunittype |  |  | 7.090 | 6 | .313 |  |  |  |
|  | Healthcarefacilityunittype(1) | -.215 | .580 | .137 | 1 | .711 | .807 | .259 | 2.515 |
|  | Healthcarefacilityunittype(2) | .325 | .582 | .311 | 1 | .577 | 1.384 | .442 | 4.331 |
|  | Healthcarefacilityunittype(3) | -2.014 | 2.331 | .746 | 1 | .388 | .134 | .001 | 12.868 |
|  | Healthcarefacilityunittype(4) | .921 | 1.480 | .387 | 1 | .534 | 2.512 | .138 | 45.667 |
|  | Healthcarefacilityunittype(5) | 1.231 | 1.237 | .991 | 1 | .320 | 3.424 | .303 | 38.648 |
|  | Healthcarefacilityunittype(6) | 1.618 | .903 | 3.210 | 1 | .073 | 5.042 | .859 | 29.590 |
|  | Typeofhealthwork |  |  | 9.306 | 6 | .157 |  |  |  |
|  | Typeofhealthwork(1) | .887 | .586 | 2.291 | 1 | .130 | 2.429 | .770 | 7.663 |
|  | Typeofhealthwork(2) | -.268 | 1.002 | .072 | 1 | .789 | .765 | .107 | 5.448 |
|  | Typeofhealthwork(3) | 1.483 | 1.017 | 2.127 | 1 | .145 | 4.406 | .600 | 32.321 |
|  | Typeofhealthwork(4) | 2.651 | 1.384 | 3.669 | 1 | .055 | 14.174 | .940 | 213.668 |
|  | Typeofhealthwork(5) | 1.940 | 2.400 | .654 | 1 | .419 | 6.961 | .063 | 768.153 |
|  | Typeofhealthwork(6) | 2.039 | .938 | 4.732 | 1 | .030 | 7.686 | 1.224 | 48.274 |
|  | exposurestatus |  |  | 19.471 | 2 | .000 |  |  |  |
|  | exposurestatus(1) | 2.257 | .512 | 19.459 | 1 | .000 | 9.558 | 3.506 | 26.058 |
|  | exposurestatus(2) | 1.011 | .545 | 3.448 | 1 | .063 | 2.749 | .945 | 7.993 |
|  | PPEuse(1) | .031 | .402 | .006 | 1 | .938 | 1.032 | .470 | 2.267 |
|  | Handwashing(1) | -.822 | .392 | 4.405 | 1 | .036 | .440 | .204 | .947 |
|  | hightouchsurfacesdecontaminatedfrequently(1) | .924 | .413 | 5.012 | 1 | .025 | 2.520 | 1.122 | 5.658 |
|  | wetmedicalmask(1) | -.670 | .513 | 1.709 | 1 | .191 | .512 | .187 | 1.397 |
|  | training(1) | .077 | .460 | .028 | 1 | .868 | 1.080 | .438 | 2.660 |
|  | Constant | 1.685 | 1.052 | 2.566 | 1 | .109 | 5.394 |  |  |
| a. Variable(s) entered on step 1: Agegroup, Maritalstatus, Typeofhealthcaresetting, family, Educationlevel, Experience, Healthcarefacilityunittype, Typeofhealthwork, exposurestatus, PPEuse, Handwashing, hightouchsurfacesdecontaminatedfrequently, wetmedicalmask, training. | | | | | | | | | |

| **Variables in the Equation** | | | | | | | | | |
| --- | --- | --- | --- | --- | --- | --- | --- | --- | --- |
|  | | B | S.E. | Wald | df | Sig. | Exp(B) | 95.0% C.I.for EXP(B) | |
|  |  |  |  |  |  |  |  | Lower | Upper |
| Step 1^a^ | Experience |  |  | 46.221 | 3 | .000 |  |  |  |
|  | Experience(1) | .802 | .491 | 2.662 | 1 | .103 | 2.229 | .851 | 5.840 |
|  | Experience(2) | -1.039 | .571 | 3.315 | 1 | .069 | .354 | .116 | 1.083 |
|  | Experience(3) | -2.877 | .462 | 38.701 | 1 | .000 | .056 | .023 | .139 |
|  | Constant | 1.732 | .160 | 117.253 | 1 | .000 | 5.652 |  |  |
| a. Variable(s) entered on step 1: Experience. | | | | |  |  |  |  |  |

| **Crosstab** | | | | | | | |
| --- | --- | --- | --- | --- | --- | --- | --- |
|  | | | Age group | | | | Total |
|  |  |  | 18-24 | 25-34 | 35-44 | 45-54 |  |
| Are you exposed to a confirmed COVID-19 patient? | Yes | Count | 17 | 51 | 9 | 1 | 78 |
|  |  | % within Are you exposed to a confirmed COVID-19 patient? | 21.8% | 65.4% | 11.5% | 1.3% | 100.0% |
|  | No | Count | 35 | 234 | 61 | 10 | 340 |
|  |  | % within Are you exposed to a confirmed COVID-19 patient? | 10.3% | 68.8% | 17.9% | 2.9% | 100.0% |
| Total | | Count | 52 | 285 | 70 | 11 | 418 |
|  |  | % within Are you exposed to a confirmed COVID-19 patient? | 12.4% | 68.2% | 16.7% | 2.6% | 100.0% |

| **Crosstab** | | | | | | |
| --- | --- | --- | --- | --- | --- | --- |
|  | | | Marital Status | | | Total |
|  |  |  | single | married | separated |  |
| Are you exposed to a confirmed COVID-19 patient? | Yes | Count | 50 | 26 | 2 | 78 |
|  |  | % within Are you exposed to a confirmed COVID-19 patient? | 64.1% | 33.3% | 2.6% | 100.0% |
|  | No | Count | 166 | 164 | 10 | 340 |
|  |  | % within Are you exposed to a confirmed COVID-19 patient? | 48.8% | 48.2% | 2.9% | 100.0% |
| Total | | Count | 216 | 190 | 12 | 418 |
|  |  | % within Are you exposed to a confirmed COVID-19 patient? | 51.7% | 45.5% | 2.9% | 100.0% |

| **Crosstab** | | | | | | | | |
| --- | --- | --- | --- | --- | --- | --- | --- | --- |
|  | | | Type of health care setting | | | | | Total |
|  |  |  | Hospital | Out patient clinic | Health center | Home care for mild cases | community pharmacy |  |
| Are you exposed to a confirmed COVID-19 patient? | Yes | Count | 68 | 1 | 6 | 2 | 1 | 78 |
|  |  | % within Are you exposed to a confirmed COVID-19 patient? | 87.2% | 1.3% | 7.7% | 2.6% | 1.3% | 100.0% |
|  | No | Count | 260 | 10 | 35 | 12 | 23 | 340 |
|  |  | % within Are you exposed to a confirmed COVID-19 patient? | 76.5% | 2.9% | 10.3% | 3.5% | 6.8% | 100.0% |
| Total | | Count | 328 | 11 | 41 | 14 | 24 | 418 |
|  |  | % within Are you exposed to a confirmed COVID-19 patient? | 78.5% | 2.6% | 9.8% | 3.3% | 5.7% | 100.0% |

| **Crosstab** | | | | | | |
| --- | --- | --- | --- | --- | --- | --- |
|  | | | range of family size | | | Total |
|  |  |  | <3 | 3-5 | >5 |  |
| Are you exposed to a confirmed COVID-19 patient? | Yes | Count | 52 | 17 | 9 | 78 |
|  |  | % within Are you exposed to a confirmed COVID-19 patient? | 66.7% | 21.8% | 11.5% | 100.0% |
|  | No | Count | 157 | 131 | 52 | 340 |
|  |  | % within Are you exposed to a confirmed COVID-19 patient? | 46.2% | 38.5% | 15.3% | 100.0% |
| Total | | Count | 209 | 148 | 61 | 418 |
|  |  | % within Are you exposed to a confirmed COVID-19 patient? | 50.0% | 35.4% | 14.6% | 100.0% |

| **Crosstab** | | | | | | | | |
| --- | --- | --- | --- | --- | --- | --- | --- | --- |
|  | | | Education level | | | | | Total |
|  |  |  | Diploma | BSc | MSc | PhD/specialist | other |  |
| Are you exposed to a confirmed COVID-19 patient? | Yes | Count | 12 | 52 | 8 | 4 | 2 | 78 |
|  |  | % within Are you exposed to a confirmed COVID-19 patient? | 15.4% | 66.7% | 10.3% | 5.1% | 2.6% | 100.0% |
|  | No | Count | 61 | 210 | 49 | 8 | 12 | 340 |
|  |  | % within Are you exposed to a confirmed COVID-19 patient? | 17.9% | 61.8% | 14.4% | 2.4% | 3.5% | 100.0% |
| Total | | Count | 73 | 262 | 57 | 12 | 14 | 418 |
|  |  | % within Are you exposed to a confirmed COVID-19 patient? | 17.5% | 62.7% | 13.6% | 2.9% | 3.3% | 100.0% |

| **Crosstab** | | | | | | | |
| --- | --- | --- | --- | --- | --- | --- | --- |
|  | | | Experience | | | | Total |
|  |  |  | 1-10 | 11-20 | 21-30 | <1 |  |
| Are you exposed to a confirmed COVID-19 patient? | Yes | Count | 46 | 5 | 5 | 22 | 78 |
|  |  | % within Are you exposed to a confirmed COVID-19 patient? | 59.0% | 6.4% | 6.4% | 28.2% | 100.0% |
|  | No | Count | 260 | 63 | 10 | 7 | 340 |
|  |  | % within Are you exposed to a confirmed COVID-19 patient? | 76.5% | 18.5% | 2.9% | 2.1% | 100.0% |
| Total | | Count | 306 | 68 | 15 | 29 | 418 |
|  |  | % within Are you exposed to a confirmed COVID-19 patient? | 73.2% | 16.3% | 3.6% | 6.9% | 100.0% |

| **Crosstab** | | | | | | | | | | |
| --- | --- | --- | --- | --- | --- | --- | --- | --- | --- | --- |
|  | | | Health care facility unit type in which the health worker works? | | | | | | | Total |
|  |  |  | Outpatient | Emergency | Medical Unit | laboratory | Pharmacy | Isolation Center | Other |  |
| Are you exposed to a confirmed COVID-19 patient? | Yes | Count | 16 | 33 | 16 | 7 | 1 | 1 | 4 | 78 |
|  |  | % within Are you exposed to a confirmed COVID-19 patient? | 20.5% | 42.3% | 20.5% | 9.0% | 1.3% | 1.3% | 5.1% | 100.0% |
|  | No | Count | 60 | 59 | 81 | 15 | 65 | 13 | 47 | 340 |
|  |  | % within Are you exposed to a confirmed COVID-19 patient? | 17.6% | 17.4% | 23.8% | 4.4% | 19.1% | 3.8% | 13.8% | 100.0% |
| Total | | Count | 76 | 92 | 97 | 22 | 66 | 14 | 51 | 418 |
|  |  | % within Are you exposed to a confirmed COVID-19 patient? | 18.2% | 22.0% | 23.2% | 5.3% | 15.8% | 3.3% | 12.2% | 100.0% |

| **Crosstab** | | | | | | | | | | |
| --- | --- | --- | --- | --- | --- | --- | --- | --- | --- | --- |
|  | | | Type of health work | | | | | | | Total |
|  |  |  | Medical doctor | nurse (midwife) | Patient transporter | health officer | Pharmacist personnel | laboratory personnel | other |  |
| Are you exposed to a confirmed COVID-19 patient? | Yes | Count | 36 | 26 | 3 | 2 | 1 | 7 | 3 | 78 |
|  |  | % within Are you exposed to a confirmed COVID-19 patient? | 46.2% | 33.3% | 3.8% | 2.6% | 1.3% | 9.0% | 3.8% | 100.0% |
|  | No | Count | 47 | 133 | 13 | 18 | 74 | 18 | 37 | 340 |
|  |  | % within Are you exposed to a confirmed COVID-19 patient? | 13.8% | 39.1% | 3.8% | 5.3% | 21.8% | 5.3% | 10.9% | 100.0% |
| Total | | Count | 83 | 159 | 16 | 20 | 75 | 25 | 40 | 418 |
|  |  | % within Are you exposed to a confirmed COVID-19 patient? | 19.9% | 38.0% | 3.8% | 4.8% | 17.9% | 6.0% | 9.6% | 100.0% |

| **Crosstab** | | | | | | |
| --- | --- | --- | --- | --- | --- | --- |
|  | | | exposurestatus | | | Total |
|  |  |  | exposed | not exposed | probably exposed |  |
| Are you exposed to a confirmed COVID-19 patient? | Yes | Count | 46 | 13 | 19 | 78 |
|  |  | % within Are you exposed to a confirmed COVID-19 patient? | 59.0% | 16.7% | 24.4% | 100.0% |
|  | No | Count | 118 | 154 | 68 | 340 |
|  |  | % within Are you exposed to a confirmed COVID-19 patient? | 34.7% | 45.3% | 20.0% | 100.0% |
| Total | | Count | 164 | 167 | 87 | 418 |
|  |  | % within Are you exposed to a confirmed COVID-19 patient? | 39.2% | 40.0% | 20.8% | 100.0% |

| **Crosstab** | | | | | |
| --- | --- | --- | --- | --- | --- |
|  | | | PPEuse | | Total |
|  |  |  | Good | bad |  |
| Are you exposed to a confirmed COVID-19 patient? | Yes | Count | 31 | 47 | 78 |
|  |  | % within Are you exposed to a confirmed COVID-19 patient? | 39.7% | 60.3% | 100.0% |
|  | No | Count | 149 | 191 | 340 |
|  |  | % within Are you exposed to a confirmed COVID-19 patient? | 43.8% | 56.2% | 100.0% |
| Total | | Count | 180 | 238 | 418 |
|  |  | % within Are you exposed to a confirmed COVID-19 patient? | 43.1% | 56.9% | 100.0% |

| **Crosstab** | | | | | |
| --- | --- | --- | --- | --- | --- |
|  | | | Hand washing behaviour | | Total |
|  |  |  | good | bad |  |
| Are you exposed to a confirmed COVID-19 patient? | Yes | Count | 52 | 26 | 78 |
|  |  | % within Are you exposed to a confirmed COVID-19 patient? | 66.7% | 33.3% | 100.0% |
|  | No | Count | 184 | 156 | 340 |
|  |  | % within Are you exposed to a confirmed COVID-19 patient? | 54.1% | 45.9% | 100.0% |
| Total | | Count | 236 | 182 | 418 |
|  |  | % within Are you exposed to a confirmed COVID-19 patient? | 56.5% | 43.5% | 100.0% |

| **Crosstab** | | | | | |
| --- | --- | --- | --- | --- | --- |
|  | | | 30. Did high touch surfaces decontaminated frequently (at least three times daily)? | | Total |
|  |  |  | Yes | No |  |
| Are you exposed to a confirmed COVID-19 patient? | Yes | Count | 20 | 58 | 78 |
|  |  | % within Are you exposed to a confirmed COVID-19 patient? | 25.6% | 74.4% | 100.0% |
|  | No | Count | 127 | 213 | 340 |
|  |  | % within Are you exposed to a confirmed COVID-19 patient? | 37.4% | 62.6% | 100.0% |
| Total | | Count | 147 | 271 | 418 |
|  |  | % within Are you exposed to a confirmed COVID-19 patient? | 35.2% | 64.8% | 100.0% |

| **Crosstab** | | | | | |
| --- | --- | --- | --- | --- | --- |
|  | | | did you remove and replace your medical mask if it became wet? | | Total |
|  |  |  | Yes | No |  |
| Are you exposed to a confirmed COVID-19 patient? | Yes | Count | 49 | 29 | 78 |
|  |  | % within Are you exposed to a confirmed COVID-19 patient? | 62.8% | 37.2% | 100.0% |
|  | No | Count | 251 | 89 | 340 |
|  |  | % within Are you exposed to a confirmed COVID-19 patient? | 73.8% | 26.2% | 100.0% |
| Total | | Count | 300 | 118 | 418 |
|  |  | % within Are you exposed to a confirmed COVID-19 patient? | 71.8% | 28.2% | 100.0% |

| **Crosstab** | | | | | |
| --- | --- | --- | --- | --- | --- |
|  | | | Did you have any episode of accidental exposure with biological fluid/respiratory secretions? | | Total |
|  |  |  | Yes | No |  |
| Are you exposed to a confirmed COVID-19 patient? | Yes | Count | 13 | 65 | 78 |
|  |  | % within Are you exposed to a confirmed COVID-19 patient? | 16.7% | 83.3% | 100.0% |
|  | No | Count | 85 | 254 | 339 |
|  |  | % within Are you exposed to a confirmed COVID-19 patient? | 25.1% | 74.9% | 100.0% |
| Total | | Count | 98 | 319 | 417 |
|  |  | % within Are you exposed to a confirmed COVID-19 patient? | 23.5% | 76.5% | 100.0% |

| **Crosstab** | | | | | |
| --- | --- | --- | --- | --- | --- |
|  | | | Have you taken any training (in-service, online.) training regarding COVID-19? | | Total |
|  |  |  | yes | No |  |
| Are you exposed to a confirmed COVID-19 patient? | Yes | Count | 35 | 43 | 78 |
|  |  | % within Are you exposed to a confirmed COVID-19 patient? | 44.9% | 55.1% | 100.0% |
|  | No | Count | 94 | 246 | 340 |
|  |  | % within Are you exposed to a confirmed COVID-19 patient? | 27.6% | 72.4% | 100.0% |
| Total | | Count | 129 | 289 | 418 |
|  |  | % within Are you exposed to a confirmed COVID-19 patient? | 30.9% | 69.1% | 100.0% |
